# Effect of Spatial Resolution on Accurate Detection and Localization of Arrhythmia Rotors in Human Right Ventricular Tachycardia

**DOI:** 10.3390/jcdd11100322

**Published:** 2024-10-12

**Authors:** Maria Inês F. Gândara, Igor R. Efimov, Kedar K. Aras

**Affiliations:** 1 Department of Biomedical Engineering, NOVA University, 1099-085 Lisbon, Portugal; inesgandara@gmail.com; 2 Department of Biomedical Engineering, Northwestern University, Chicago, IL 60208, USA; igor.efimov@northwestern.edu; 3 Department of Medicine, Northwestern University, Chicago, IL 60611, USA; 4 Department of Physiology and Biophysics, University at Buffalo, Buffalo, NY 14203, USA; 5 Department of Biomedical Engineering, University at Buffalo, Buffalo, NY 14228, USA

**Keywords:** arrhythmias, rotor detection, rotor localization, spatial resolution, donor human hearts, ventricular arrhythmias

## Abstract

The goal of this study was to identify the spatial resolution requirements for accurate rotor detection and localization in human right ventricular tachyarrhythmias. Poor spatial resolution is often cited as a reason for the inaccuracy of cardiac mapping catheters in detecting and localizing arrhythmia rotors. High-resolution (0.7 mm) arrhythmia data from optical recordings obtained from human donor hearts (n = 12) were uniformly downsampled to lower resolutions (1.4–7 mm) to approximate the spatial resolution (4 mm) of clinical mapping catheters. Rotors were tracked at various subresolutions and compared to the rotors in the original data by computing F1-scores to create accuracy profiles for both rotor detection and localization. Further comparisons were made according to arrhythmia type, donor sex, anatomical region, and mapped surface: endocardium or epicardium. For a spatial resolution of 4.2 mm, the accuracies of rotor detection and localization were 57% ± 4% and 61% ± 7%, respectively. Arrhythmia type affected the accuracy of rotor detection (monomorphic ventricular tachycardia, 58% ± 4%; ventricular fibrillation, 56% ± 8%) and localization (monomorphic ventricular tachycardia, 70% ± 4%; ventricular fibrillation, 54% ± 13%). However, donor sex, anatomical region (right ventricular outflow tract, mid, and apical), and mapped surface (epicardium and endocardium) did not significantly affect rotor detection or localization accuracy. To achieve rotor detection accuracy of 80%, a spatial resolution of 1.4 mm or better is needed. The accuracy profiles provided here serve as a guideline for future mapping device development.

## 1. Introduction

Ventricular tachyarrhythmias are responsible for at least 300,000 sudden cardiac deaths in the United States [1]. These arrhythmias present as ventricular tachycardia (VT) or ventricular fibrillation (VF) and arise from either abnormal impulse formation (focal sources) or abnormal conduction (reentrant rotors) [2,3,4].

Electrotherapy and ablation therapy remain the primary treatment strategies for VT/VF [5,6,7,8]. Ablation therapy has had varied success [9,10,11,12], with insufficient resolution and electrode density of current cardiac mapping devices cited as reasons for limited accuracy in detecting rotor ablation targets. Even so, current cardiac mapping catheters do not provide sufficient rotor detection or localization accuracy because of the low spatial resolution, which can result in detection errors, such as missing or mislocating rotors or false-positive detection. These errors can lead to erroneous ablation of healthy tissue and failure to apply therapy where it is needed. Leads of implantable cardioverter defibrillators used in electrotherapy are also limited by low resolution for sensing VT/VF, reducing their ability to detect and localize arrhythmia rotors. Thus, high-resolution tracking of arrhythmia rotors would benefit both therapies.

There is a need to establish a minimum spatial resolution to accurately detect and locate rotors to improve mapping devices and ablative or electrotherapy outcomes. Here, we characterized the loss of arrhythmia rotor information when low electrode resolution is used to map cardiac arrhythmias. Specifically, we downsampled high-resolution optical maps of tachyarrhythmias in donor human hearts to available catheter electrode spatial resolutions.

## 2. Methods

Full method details are available in the Supplementary Materials.

### 2.1. Donor Heart Data

All data are from the optical and electrical mapping studies of de-identified donor human hearts conducted as part of a different study [13]. Briefly, de-identified donor human hearts (procured from Washington Regional Transplant Community, Washington, DC, USA) were arrested using ice-cold cardioplegia and transported to the laboratory (within 2–4 h after heart explantation) for right ventricle (RV) electrophysiology studies. Tissue was prepared by dissecting the aorta and pulmonary artery to expose and isolate the right and left coronary arteries for cannulation. Most of the atria, left ventricle, and posterior RV were removed to expose the anterior RV, including the right ventricle outflow tract. The resulting RV tissue preparation was suspended vertically in a bath to allow dual sided (epicardial and endocardial) optical and electrical mapping experiments. Arrhythmia was induced using a combination of isoproterenol (100 nM) followed by S1S1 restitution protocol or burst pacing (50 Hz). All arrhythmias induced by S1S1 pacing or burst pacing were sustained for at least 15 min. Epicardial and endocardial optical action potentials and electrograms recordings during VT/VF from the right ventricles of 12 donor human heart data sets (6 male, 6 female) were analyzed (Supplementary Tables S1 and S2) for this study.

### 2.2. Arrhythmia Data Processing and Analysis

**Figure 1:**
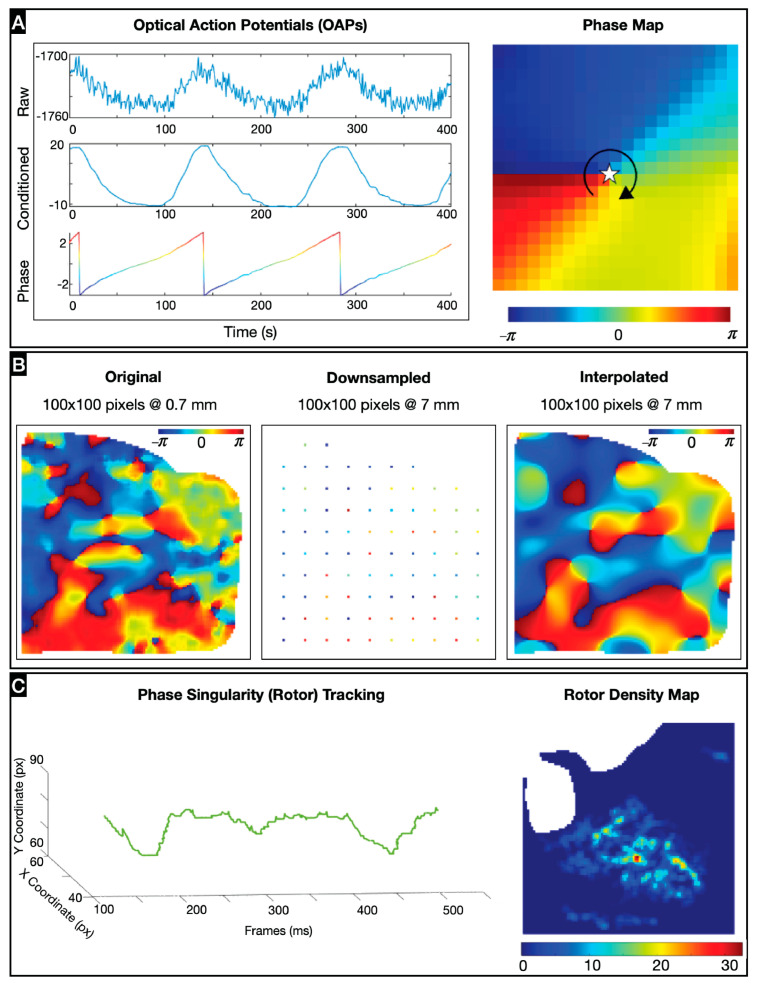
Data processing and analysis: (**A**) Signal conditioning of representative optical action potentials and conversion to phase map with an example phase singularity (rotor), identified as star with the arrow representing the rotor direction. (**B**) Downsampling of original optical mapping data followed by upsampling using cubic spline interpolation (**C**) Metrics for evaluating arrhythmia dynamics, including spatiotemporal tracking of rotors and rotor peak density maps.

All signal processing was performed in MATLAB version 2021a. The optical action potentials (spatial resolution, 0.7 mm) were conditioned with a combination of spatial binning (5-by-5 pixel bin), powerline noise removal at 60 Hz, low-pass filtering at 100 Hz, and baseline drift correction. The optical action potentials were converted to phase signals using the Hilbert transform (Figure 1A, Figures S1 and S2). To simulate different spatial resolutions, the phase maps were gradually downsampled to nine subresolutions ranging uniformly from 1.4 to 7.0 mm. Afterward, the decimated maps were upsampled back to the original resolution with cubic splice interpolation to directly compare resolutions (Figure 1B).

Phase singularities (PSs), or rotors, are identifiable as points on a phase map where the range of cardiac excitation values merge, and these heterogeneities in the impulse conduction were detected with the Sobel edge detection operator performed in the *x* and *y* directions. Rotors were tracked in time by connecting PSs that were within a 2-pixel radius from each other and ≤5 ms apart (Supplementary Figures S3–S6). To eliminate noise, only rotors lasting ≥50 ms were considered. Stable rotors were those that lasted at least the minimum cycle length (CL) of the recording. Rotor density maps were created to identify spatial incidence clusters for each resolution (Figure 1C, Figures S7 and S8). The maps were obtained by summing the rotors’ occurrences at each pixel and spatially smoothing the resulting images with a 3-by-3 pixel bin. To find the density peaks and ignore misleading peaks, a threshold of 75% of the highest density value was imposed.

Rotors and rotor density peaks for the different subresolutions and the original were matched. For rotors to match, they had to have the same chirality, share at least one-quarter of their lifespan, and have an average spatial displacement of ≤5 pixels (3.5 mm). Cases where a single rotor appeared fragmented into shorter ones for lower resolutions were considered. Density peaks were matched if they were within a certain radius of each other, with different radii tested (1.4 mm, 2.1 mm, and 3.5 mm).

To verify the matches for each subresolution, two metrics were calculated: precision (ratio of true rotors found to all the rotors found in a subresolution) and recall (ratio of true rotors found in a subresolution to all rotors found in the original resolution). The harmonic mean of the two metrics (F1-score) was tabulated. The F1-score of the tracked rotors in subresolutions served as a measure of rotor detection accuracy, and the F1-score of the rotor density peaks detected in subresolutions was a measure of rotor localization accuracy. Rotor incidence and mean rotor duration for each recording were also extracted to analyze arrhythmia dynamics. The results for arrhythmia type, donor sex, anatomical region, and mapped surface were compared in a categorical analysis.

This work assumes that the fluorescence data obtained from optical mapping directly correlate with electrical data. Simultaneous electrical and optical mapping data were only available for one data set but confirmed a strong correlation between electrical and optical cardiac mapping data (Supplementary Figures S9–S12).

### 2.3. Statistical Analyses

Data were analyzed using GraphPad Prism version 9.2.0 for Windows. The samples presented are the averages from all recordings for each donor heart. Average trends across the cohort of donor hearts are reported as sample means ± SEMs. Repeated measures ANOVA were used for spatial resolution comparisons (one-way) and spatial resolution comparisons between groups (by arrhythmia type, donor sex, anatomical region, and mapped surface). When relevant, post hoc multiple comparisons tests were performed to compare between arrhythmia type, mapped surface, and donor sex (Šidak’s), and between anatomical regions (Tukey’s). Dunnett’s test was used when comparing group means for each subresolution against the ground truth for rotor incidence and duration. When values were missing, a mixed-effects analysis was performed. Significance was defined as a *p*-value of <0.05.

## 3. Results

### 3.1. Effect of Methodology on Rotor Matching

**Figure 2:**
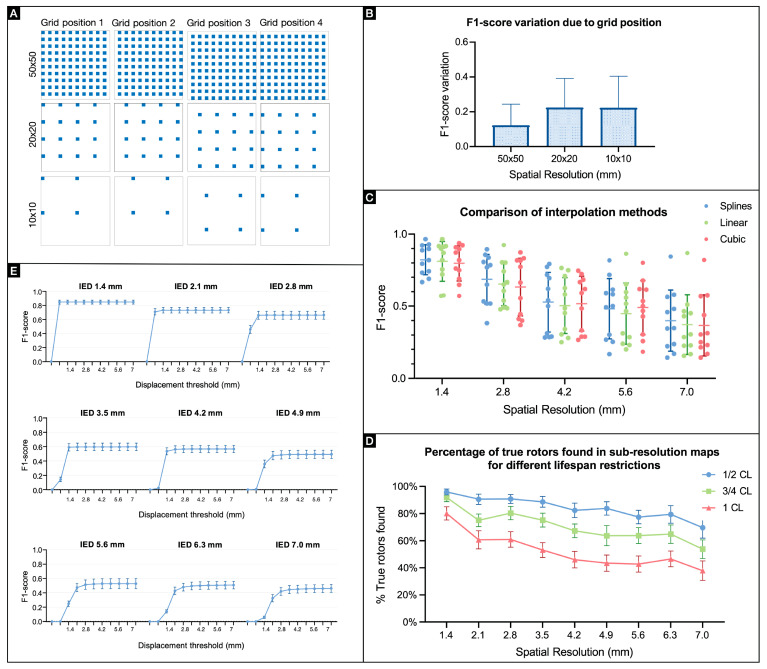
Effect of methodology on rotor correlation: (**A**) Representative matrices of downsampling grid positions tested at one-half, one-fifth, and one-tenth of the original resolution. (**B**) F1-score variation induced by the position of the downsampling grid (n = 11); data means ± SDs. (**C**) F1-scores for different interpolation methods. (**D**) Percentages of true rotors found at subresolutions for different lifespan restrictions. (**E**) F1-score profiles for different spatial displacement thresholds (n = 11); data are means ± SEMs.

The algorithms for processing arrhythmia data and matching PSs at different resolutions include variables that can inherently impact the results (F1-scores). We therefore evaluated how downsampling, interpolation techniques, and algorithm thresholds for matching (rotor duration, rotor location) impact F1-scores (Figure 2).

When downsampling, only a proportion of the recorded signals are used for the analysis. The signals selected were uniformly spaced in a grid and the position remained fixed throughout the study. To assess uncertainty introduced by the grid position, we tested four positions—default, shift right, shift down, and shift down and left (Figure 2A)—for three subresolutions (D = {2, 5, 10}). The F1-score was obtained for each position, and the variation among the values was computed for each recording (Figure 2B). These values represented variations of 10% to 20% of the F1-score, suggesting that the position of the electrode may influence rotor mapping results.

We used three different interpolation techniques (linear, cubic, and cubic spline) to evaluate whether the algorithm for interpolating downsampled data introduced variation. The F1-scores obtained with each technique were compared for five subresolutions (Figure 2C); F1-scores obtained with three techniques did not differ significantly (two-way repeated measures ANOVA, *p* = 0.1712).

When tracking rotors, the PS lifespan (rotor duration) threshold was set to distinguish between unstable and stable rotors; only stable rotors were analyzed to calculate F1-scores. A loss in spatial resolution can lead to fragmentation of the PS path, and thus the algorithms will not be able to track them across their lifespan. To account for a potential type II error (false negative) associated with PS path fragmentation, the number of true rotors for lower resolutions was investigated for different lifespan restrictions (1 CL, ½ CL, and ¾ CL) (Figure 2D). The percentage of true rotors found was higher for ½ CL than ¾ and 1 CL, suggesting a stronger correlation between shorter-lasting PSs in subresolution maps and the stable rotors at the original resolution.

The associations between the PSs found in the original maps and those at lower resolutions were also constrained by how close the PSs in subresolution maps had to be to the original location (spatial accuracy threshold). To analyze how PS detection was affected by this displacement restriction, an F1-score profile was created for each subresolution with accepted displacement ranging from 0 to 7 mm (Figure 2E).

These graphs show that a plateau is reached for every subresolution. The spatial accuracy threshold at which the plateau is reached depends on the spatial resolution (i.e., IED). For the highest IED considered, 7.0 mm, the minimum spatial accuracy radius (or displacement threshold) for rotor detection was 3.5 mm, which was selected as the default spatial accuracy threshold for this study.

### 3.2. Effect of Spatial Resolution on Arrhythmia Dynamics

**Figure 3:**
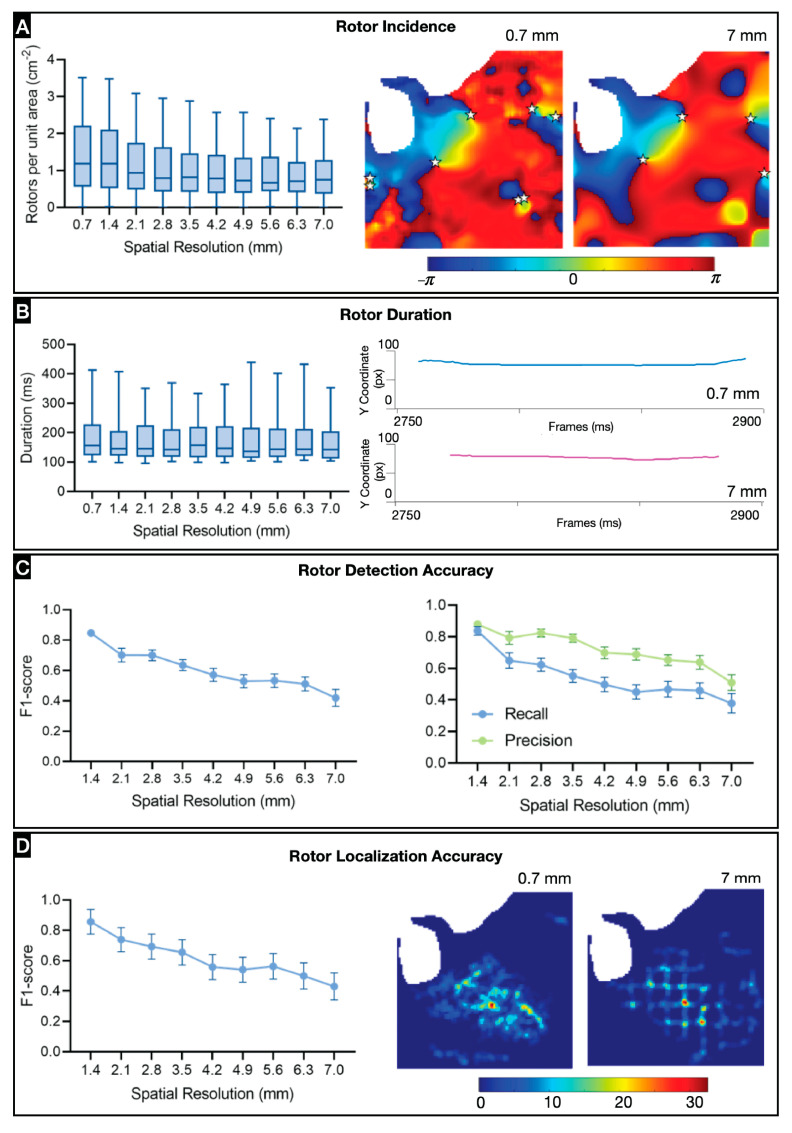
Effect of spatial resolution on arrhythmia dynamics: (**A**) Stable rotor incidence at different spatial resolutions (n = 12). Right, representative phase maps of rotor (displayed as stars) incidence at original (0.7 mm) and one-tenth resolution (7 mm). (**B**) Duration of stabler rotors at different spatial resolutions. Right, representative example of impact of the impact of lower resolution on rotor duration. (**C**) Stable rotor detection accuracy at different spatial resolutions, given by F1-score of rotor detection. Right, precision and recall values at different spatial resolutions. (**D**) Rotor localization accuracy given by F1-score of rotor density peaks for stable rotors. Right, representative rotor density maps of a single VF recording for two different spatial resolutions.

Arrhythmia dynamics are measured as rotor incidence, rotor duration, rotor detection, and rotor localization. Rotor Incidence reflects the level of organization of arrhythmias, as more disorganized arrhythmias are expected to result in higher incidences of PS (both stable and unstable). The incidence of stable and unstable rotors decreased with resolution and stabilized at resolutions lower than 2.1 mm (Figure 3A), suggesting that most stable rotors are detected even at low resolutions. The rotor incidence at each subresolution was compared to that at the original resolution (0.7 mm); all but the incidence at 1.4 mm resolution were significantly different from that at 0.7 mm (Supplementary Figure S13).

Rotor duration is an important characteristic because longer-lasting rotors are likely more stable. Stable rotor duration did not differ significantly at different resolutions (Figure 3B), suggesting that rotor duration is independent of spatial resolution. However, there were differences when both stable and unstable rotors were considered; durations at the original resolution were significantly different from those at subresolutions of 2.1–5.6 mm (Supplementary Figure S14), which may reflect an interpolation artifact. As the amount of actual data decreases (i.e., with lower resolution), the interpolation effect amplifies, such that the rotors are attributed to discrete locations, which could lead to an artificial increase in rotor duration.

Rotor incidence cannot be used to determine if the same rotors are being detected at lower resolutions as at the original one. Thus, we computed F1-scores for each subresolution (Figure 3C and Figure S15). The F1-score provides a single measure summarizing the accuracy of detection, considering precision and sensitivity (recall). We compared the precision and sensitivity to determine which impacts the overall accuracy the most. Recall values are lower than for precision, suggesting that it is more likely that rotors are being lost at low resolutions than being falsely detected at high resolutions.

Longer-lasting stable rotors may be more relevant to the arrhythmia dynamics, and to therapy, thus we sought to determine if they are being recognized. We computed the F1-score for different stable rotor duration thresholds and found that rotors were detected with similar accuracy up to an IED of 2.1 mm (Supplementary Figure S16).

Lastly, we assessed rotor localization accuracy using two metrics: displacement between associated PS paths and the F1-score for PS density map peaks. The mean and maximum displacement of PSs increased as resolution decreased (Supplementary Figure S17). Density maps do not analyze PSs individually but instead provide a global picture of their spatial distribution. This is particularly helpful to determine if the correct location is being selected for therapy, even if the true rotors are not consistently being tracked. Rotors tend to cluster in regions, and these regions may be identifiable even when individual PSs are not detectable at lower resolutions, as seen in the representative stable rotor density map generated from a VF instance (Supplementary Figure S18). To determine the effect of spatial resolution on PS density peaks, we computed F1-scores for peak detection at different subresolutions (Figure 3D and Figure S19). The F1-score for rotor localization decreased with loss in spatial resolution, but this was not statistically significant. Unstable rotors have a higher incidence as compared to the incidence of stable rotors. Therefore, the density peaks of unstable rotors overshadowed that of stable rotor peaks, resulting in missed density peaks of stable rotors (Supplementary Figure S20).

### 3.3. Effect of Spatial Resolution on Arrhythmia Type and Location

**Figure 4:**
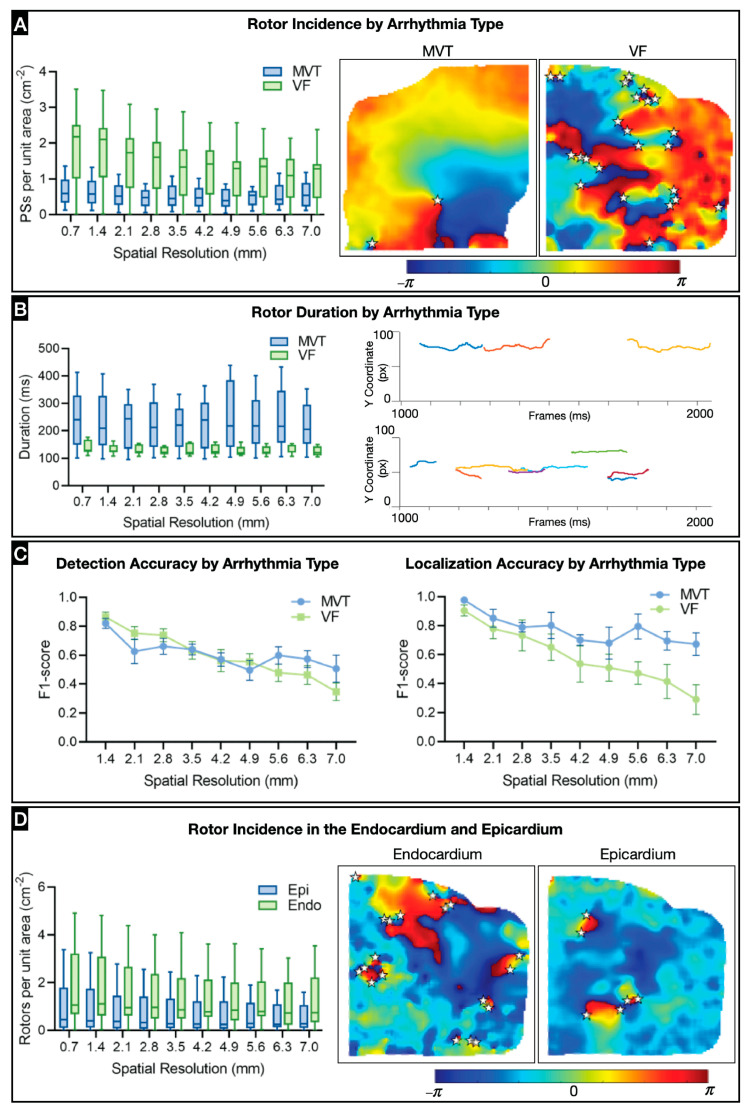
Effect of spatial resolution on arrhythmia type and location: (**A**) Comparison of stable rotor incidence between monomorphic ventricular tachycardia (MVT; n = 5) and ventricular fibrillation (VF; n = 6). Right, representative examples of MVT and VF phase maps with rotors (displayed as stars). (**B**) Comparison of stable rotor duration between MVT and VF. Right, representative examples of stable MVT and VF rotors tracked over time. (**C**) Comparison of rotor detection and localization accuracy between MVT and VF. (**D**) Comparison of stable rotor incidence in epicardium (Epi) and endocardium (Endo). Right, representative examples of epicardial and endocardial phase maps with rotors (displayed as stars) from a VF recording.

Arrhythmia dynamics vary drastically between stable (MVT) and unstable (VF) arrhythmias. We evaluated these separately to determine how their measurements are affected by spatial resolution. For rotor incidence, the difference between MVT and VF was not statistically significant; however, there was a tendency for more rotors to be detected during VF (Figure 4A and Figure S21). The variation due to spatial resolution disproportionately affected VF more than MVT because of the high rotor incidence and low rate of rotor stability for VF. Indeed, MVT is characterized by long-lasting stable rotors, which are easier to track, even at low resolutions. In the case of VF, except for an IED of 1.4 mm, all subresolutions had a significantly lower incidence (Supplementary Figures S22 and S23).

As expected, MVT rotors lasted longer than VF rotors (*p* = 0.0286), and the difference in duration was approximately the same for every spatial resolution, indicating that the spatial resolution does not affect rotor duration (Figure 4B and Figure S24). There was no significant difference between F1-scores for MVT and VF detection accuracy (Figure 4C and Figure S25).

There were no sex differences in rotor incidence, rotor duration, rotor detection, or rotor localization accuracy (Supplementary Figure S26). However, the F1-scores for rotor detection and rotor localization consistently trended lower in females than in males.

Anatomical differences in arrhythmia dynamics were also evaluated with the field of view divided into the upper right ventricular outflow tract (well-documented source of arrhythmogenic triggers), mid-ventricular region, and lower apical region (Supplementary Figure S27). However, there were no statistically significant differences across the three regions relative to rotor incidence, rotor duration, or rotor detection accuracy.

We also compared differences in arrhythmia dynamics between epicardial and endocardial surfaces (Figure 4D, Figures S28 and S29). Rotor incidence was higher in the endocardium than in the epicardium. Moreover, rotor incidence in the endocardium decreased as the resolution was lost, possibly because the epicardial adipose tissue blocks the underlying myocardial tissue. There were no significant differences between epicardial and endocardial arrhythmia dynamics relative to rotor duration, rotor detection accuracy, or rotor localization accuracy.

### 3.4. Spatial Resolution for Accurate Rotor Detection and Localization

**Figure 5:**
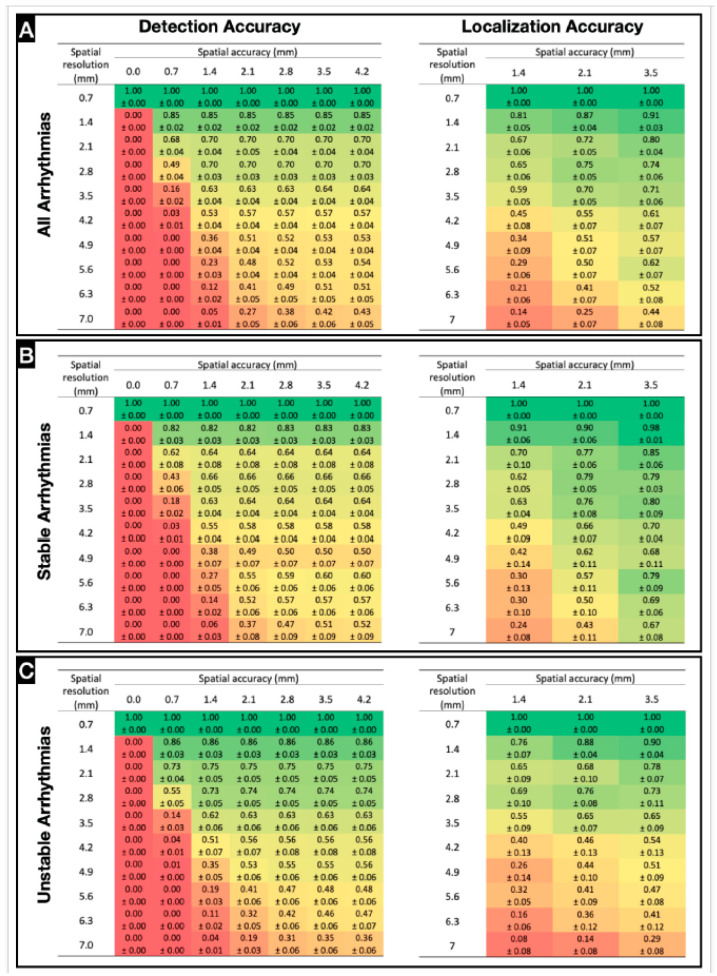
Spatial resolution for accurate rotor detection and localization: Color coded rotor detection and localization accuracy profiles for multiple spatial resolution and spatial accuracies for all arrhythmias (n = 11) (**A**), for stable arrhythmias (MVT) (**B**), and for unstable arrhythmias (VT) (**C**). Color coded from red (zero) to green (one).

Spatial accuracy is critical for determining the minimum spatial resolution to map arrhythmia drivers. We created accuracy profiles for stable rotor detection and localization considering both spatial resolution and spatial accuracy radius (Figure 5A and Figure S30A,D).

If the average IED for current cardiac mapping catheters is ~4 mm (not considering distances between splines for basket catheters) (Supplementary Table S3), the expected detection accuracy is 57% ± 4%. There is a 95% probability that the accuracy mean for an IED of 4.2 mm does not reach ≥70%. Thus, an F1-score of 80% (a good localization accuracy) can be reached with a spatial resolution of 2.1 mm. Currently, the clinical resolution is ~4.2 mm, with a localization accuracy of only 61% ± 7%.

Rotor detection accuracy for stable arrhythmias (MVT) (Figure 5B and Figure S30B,E) initially drops as the spatial resolution is lost. However, accuracy stabilizes at 50–65% for IED ≥ 2.1 mm. For a resolution of 4.2 mm (clinical spatial resolution average), the rotor detection accuracy is 58% ± 4% (95% confidence interval, 45–70%) for a spatial accuracy of 3.5 mm. For this value to be ≥80% or to increase reasonably, the spatial resolution must be at least 1.4 mm. Localization accuracy values are higher for MVT than for VF. For spatial accuracy of 3.5 mm, the average localization accuracy is not ≤67%. For resolutions of ≤2.8 mm mean accuracy varies between 65% and 80%. For the average current clinical resolution, localization accuracy is 70% ± 4% (95% confidence interval, 60–80%).

For VF (Figure 5C and Figure S30C,F), the minimum spatial resolution for an accuracy of >80% is 1.4 mm, the same as for MVT and for all recordings. For a clinical resolution of ~4 mm, rotor detection accuracy is 56% ± 8%. For the lowest spatial resolution of 7 mm, rotor detection accuracy reaches 36% ± 6%. For a spatial accuracy of 3.5 mm, the spatial resolution must be at least 4.9 mm for a mean localization accuracy to be ≥50%. To be ≥70%, the resolution must be ≤2.8 mm, and to reach 90%, it must be at least 1.4 mm. For current clinical resolution, localization accuracy is 54% ± 13%.

## 4. Discussion

In this study, we analyzed the effects of spatial resolution (IED) on mapped arrhythmia dynamics (rotor incidence and rotor duration) and created accuracy profiles for rotor detection and rotor localization at different spatial resolutions. We used high-resolution optical and electrical mapping data from donor human hearts from previous studies and confirmed that optical and electrical data match. Based on F1-scores, we found that the accuracies of detecting and localizing rotors at current clinical catheter mapping resolution (~4.2 mm) were 57% ± 4% and 61% ± 7%, respectively. To achieve a rotor detection accuracy of at least 80%, the spatial resolution needs to be at least 1.4 mm. The accuracies were affected by arrhythmia type (MVT vs. VF), but not other factors, such as sex, anatomical region, or the surface mapped. This is an important finding that can influence the evaluations of the success/failure of ablation therapy and electrotherapy in cardiac arrhythmia patients and provides the framework for spatial resolution requirements for future catheter mapping devices, which in turn could improve therapy outcomes.

Spatial resolution requirements have been extensively investigated in simulation studies for atrial fibrillation [14,15,16,17,18]. Martinez-Mateu et al. used a virtual model of atria to determine the accuracy of simulated basket catheters to detect rotors to guide ablation and recommended a basket catheter (16 splines with 16 electrodes along each spine) with an IED of 2.4 mm along the splines and a maximum IED of 5.85 mm between splines for ablation accuracy [14]. Alessandrini et al. found that a catheter with an IED of 3 mm can locate rotors with an error within the ablation lesion dimension [15]. They also noted that currently available basket catheters do not have sufficient resolution for accurate rotor detection. Aronis et al. found that an IED of 4 mm is adequate for a localization error to fall within the lesion area, even with multiple rotors present [16]. Roney et al. looked at resolution requirements for accurate rotor and focal source identification as a function of wavelength using simulated data [17]. To test the resolution requirements, their simulated data were downsampled to increase the IED from 0.1 mm to 1–25 mm, ensuring that the positions of the data points were as close as possible to the rotor at the original full resolution. They found that IED needed to be at least 3.1 mm to correctly identify rotors and avoiding false detections. They concluded that basket catheters have adequate resolution to detect rotors, but not to rule out false ones. King et al. performed optical mapping studies on rabbit hearts and recommended an IED of 2 mm for rotor detection [18].

Most studies on spatial resolution requirements for rotor detection, including those described above, relied on cardiac simulation data, which, while useful, do not produce results as reliable as those from real mapping data. However, the use of clinical data can also be problematic, as it does not allow for effective testing at different resolutions. Animal studies are similarly inadequate; although some animal species have comparable cardiac physiology, the results from them may not always translate to human settings and the resolution of animal mapping systems is comparable to that of the clinical mapping. The study presented here overcomes these limitations, as we used data from optically mapped donor human hearts.

While MVT typically involves a macro-reentrant circuit with discrete isthmus or slow zone in the left ventricle, sustained MVT can originate in the RV [13,14]. Moreover, in terms of arrhythmia rotor complexity, MVT and VF represent the two ends of the spectrum. Thus, analyzing the MVT and VF episodes provided adequate range of arrhythmia complexity to determine the minimum electrode resolution for rotor detection and localization. Generally, low spatial resolution resulted in lower perceived rotor incidence, which may lead to misinterpretation of the mechanisms and complexity of arrhythmias. Rotor detection and localization also steadily decreased with loss of spatial resolution. Because values for recall are lower than precision values, it is more likely that rotors are being lost at lower resolutions than false rotors are being generated. Therefore, although the loss in resolution does not lead to appearance of phantom rotors, the failure to detect real ones is troubling. Notably, the measures of rotor duration were not dependent on spatial resolution.

Our results highlight the marked difference between stable and unstable arrhythmias in rotor dynamics; the more stable an arrhythmia is, the more likely it has fewer but longer-lasting rotors. The dynamics of unstable rotors seem more chaotic, appearing as several short-lived meandering rotors that lose complexity as spatial resolution decreases. By contrast, stable rotors are less affected by spatial resolution, minimizing the potential for arrhythmia mechanisms to be misinterpreted and inadequately treated. The localization accuracies of stable and unstable arrhythmias were similar up to a resolution of 2.8 mm; at lower resolutions, the peak detection accuracy was lower for unstable arrhythmias. This was not surprising because the rotors for stable arrhythmias are clustered and thus easier to localize than shorter dispersed rotors for unstable arrhythmias. If the goal is to detect stable arrhythmias, lower resolutions with a detection accuracy of ~50–60% may suffice. Resolution is more important, however, for detecting highly unstable arrhythmias.

Despite profound differences in physiological mechanisms, anatomic, and genetic determinants, and etiologies of various VT/VF, electrotherapy and catheter ablation therapy remain the only effective treatment strategies. While conventional implantable cardioverter defibrillators (ICD) can successfully terminate VT episodes, they are limited in their ability to map or sense VT/VF due to fixed leads, typically on the right ventricular endocardium and cannot accommodate multipoint or targeted pacing. In addition, the current lead ICD technology has low VT/VF sensing resolution, which can result in inappropriate shock therapy resulting in adversely affect health outcomes, quality of life, mortality [19,20], and in certain cases can cause direct fatal arrhythmias [21,22]. The limited spatial resolution of current ICD technology prevents the direct translation of our findings to existing ICD devices. However, the development of flexible and conformal electronics as a platform for designing the next generation mesh-type soft devices that can cover the entire ventricle to achieve high-density multichannel recording and deliver electrotherapy with precision is an active research area [23,24,25,26,27].

While catheter ablation has recently emerged as a preferred treatment, accurately identifying the arrhythmogenic substrate and effectively ablating all inducible VT re-entry circuits remains challenging. Catheter mapping techniques routinely used for rotor detection during VT ablation therapy include activation mapping, pace mapping, entrainment mapping, and substrate mapping [8]. In addition, electroanatomical mapping (EAM), which enables direct visual correlation between anatomical structures and electrogram morphology to help delineate areas of scarred myocardium (by using local abnormal ventricular activities including fractionated, split, low-amplitude/long-lasting, pre-systolic, and late potentials) is often employed to help improve rotor detection accuracy [28,29,30]. Our study using optical mapping data relied on phase mapping analysis to identify arrhythmia rotors and did not employ EAM, which could have provided additional insights into the myocardial substrate and the origin of arrhythmia rotors.

### Study Limitations

A considerable limitation of this study was the absence of rotors in the area mapped by the electrode array. Although the electrical and optical data correlated, further validation is needed to correlate rotor detection with electrical data and with optical data downsampled to a similar resolution. Regarding the conditioning of the data and rotor tracking algorithm, there were several steps involved that could introduce uncertainty. In this study, the positions of downsampling grid introduced variation in the F1-scores. A deeper analysis of the variability induced by data conditioning should be performed. For example, simulated data could be run through the algorithm pipeline to quantify methodological errors. Furthermore, the data are biased to a resolution of 0.7 mm, which was set as the ground truth. Future studies should compare the present results with those with optical data from a smaller field of view, which would have higher ground truth spatial resolution. In that case, however, the smaller area mapped could also influence the results. Regarding the data analysis, the F1-score was used to evaluate the accuracy of detecting and localizing rotors. However, this metric does not consider the number of false positives when no true positives are detected. This is limiting because the amount of false rotors/density peaks is still relevant when no true rotors/density peaks are detected. This was not a factor in the present study but should be considered in subsequent works. The only pseudo-electrode configuration tested was a uniform grid. Additional studies should experiment with other configurations to mimic specific mapping devices. Our analysis was constrained to right ventricular arrhythmia data [13]. As such, we cannot extrapolate the conclusions from this study to represent left ventricular arrhythmias. Moreover, the data were procured from donor hearts with co-morbidities (e.g., obesity, CAD), but lacked significant cardiomyopathy, which could limit the generalization of the study findings to diseased myocardium. While the etiology of left and right ventricular arrhythmias may vary, we speculate that the next generation ablation catheters and cardiac implantable electronic devices are likely to use similar algorithms for arrhythmia wavefront detection and rotor localization. Moreover, we have an ongoing study to evaluate arrhythmia susceptibility in the human left ventricle and to validate the findings from the current study, and plan to publish the results soon.

While optical mapping captures simultaneous activation across the myocardium, the sequential nature of clinical catheter mapping as well as short duration electrogram recordings combined with low spatial resolution can pose challenges in interpretation of individual electrograms obtained at different time points, particularly regarding detection and localization of unstable and meandering rotors. In addition, the electrogram signals obtained during intracardiac mapping may be affected by catheter orientation relative to the underlying cardiac tissue, randomly distributed with clustering of points in specific areas, and include far field data from other structures [31]. As such, the validation of our findings when considering these complexities remains to be investigated. As mapping catheters continue to technologically advance to incorporate high-density and conformal electronics, it is expected that some of the current clinical limitations may be overcome.

Optical mapping remains the gold standard for arrhythmia analysis on account of its high spatiotemporal resolution and high-quality signals for rotor detection and localization. However, direct clinical translation of the identified minimal spatial resolution derived from optical mapping studies is restricted given the differences in current mapping techniques and resolution capabilities. Advances in motion tracking techniques and innovation in optoelectronics are enabling the creation of implantable cardiac pacemakers and defibrillators with closed-loop high-resolution opto-electrical systems [32]. In addition, the emergence of optogenetics in combination with optical mapping techniques (e.g., near-infrared in vivo optical mapping) is further narrowing the gap for clinical use. The additive approaches to VT/VF ablation incorporating functional imaging in combination with high density opto-electrical mapping provides promising potential to improve the sensitivity and specificity of rotor detection and localization in a clinical setting.

## 5. Conclusions

Ablation and electrotherapy of cardiac right ventricular arrhythmias could potentially achieve higher efficacy with accurate rotor detection and localization. Our study showed that to achieve a rotor detection accuracy of 80%, a spatial resolution of at least 1.4 mm is needed, much higher than what is currently available in clinical settings. The accuracy profiles provided in this study can serve as a guideline for future mapping device development.

## 6. Perspectives

### 6.1. Clinical Competencies

Ablation therapy and electrotherapy are constrained by insufficient resolution and electrode density of current cardiac mapping catheters and ICDs, reducing their ability to detect and localize arrhythmia rotors accurately. In this study we show that to achieve a rotor detection accuracy of 80%, the spatial resolution needs to be at least 1.4 mm, much higher than what is currently available in clinical settings.

### 6.2. Translational Outlook

Ablation- and electrotherapy-based treatments of cardiac arrhythmias could achieve higher efficacy with accurate rotor detection and localization. The accuracy profiles provided in this study can serve as a guideline for future mapping device development.

## Data Availability

Data is contained within the article and Supplementary Materials.

## Supplementary Materials

The following supporting information can be downloaded at: https://www.mdpi.com/article/10.3390/jcdd11100322/s1, including detailed description of the methods, Supplementary Tables (S1–S3), and Supplementary Figures (S1–S30).

## Abbreviations

CL	cycle length
IED	inter-electrode distance
MVT	monomorphic ventricular tachycardia
PS	phase singularity
VT	ventricular tachycardia
VF	ventricular fibrillation
SR	stable rotor
