# Supplementary material for: Effect of Spatial Resolution on Accurate Detection and Localization of Arrhythmia Rotors in Human Right Ventricular Tachycardia
Source: J Cardiovasc Dev Dis. 2024 Oct 12;11(10):322. doi: 10.3390/jcdd11100322 (PMC11508746; doi:10.3390/jcdd11100322)
Supplement: Supplementary file 1 [file jcdd-11-00322-s001.zip › jcdd-3181442-supplementary.pdf]

Supplementary Materials for

Effect of Spatial Resolution on Accurate Detection and Localization of Arrhythmia Rotors in Human Right Ventricular Tachycardia

Maria Inês F. Gândara[1], Igor R. Efimov[2,3], Kedar K. Aras[4,5]

[1]Department of Biomedical Engineering, NOVA University, 1099-085 Lisbon, Portugal;

inesgandara@gmail.com

[2]Department of Biomedical Engineering, Northwestern University, Chicago, IL 60208, USA;

[3]Department of Medicine, Northwestern University, Chicago, IL 60611, USA;

Igor.efimov@northwestern.edu

[4]Department of Physiology & Biophysics, University at Buffalo, NY 14203, USA;

[5]Department of Biomedical Engineering, University at Buffalo, NY 14228, USA

Correspondence: aras@buffalo.edu; Tel: 716-887-2930

This PDF includes:

Detailed description of the methods

Supplementary Tables S1, S2, and S3

Supplementary Figures S1 - S30

Methods

The data, analytic methods, and study materials will be made available for other researchers upon reasonable request for the purposes of reproducing the results or replicating the procedure.

Data description

We analyzed the different arrhythmic episodes (MVT and VF) in the optical and electrical mapping data recorded from the donor human hearts (Supplementary Table 1 and 2).

Optical data signals (optical action potentials) were obtained at a sampling frequency of 1kHz from CMOS cameras (SCIMedia, Costa Mesa, CA) with a resolution of 100 by 100 pixels and a field of view of approximately 7.0 by 7.0 cm, resulting in a spatial resolution of 0.7 mm for the optical data.

Electrical data signals (electrograms) were obtained at a sampling frequency of 1kHz using a 64-electrode array with dimensions of 4cm by 3 cm and approximate interelectrode distance of 4 mm horizontally and 3 mm vertically, covering a total area of 12 cm^2.

Optical data processing

All data processing was performed in custom MATLAB software CADENCE (https://github.com/kedararas/Cadence).

Data filtering

Raw optical data were preprocessed via the following steps:

- Spatial binning: The data were spatially smoothed to decrease noise by replacing each pixel with the average of itself and its neighbors. To that end, the data matrix was convoluted with a matrix of ones (MATLAB function "conv2"), whose size depended on the number of neighbors to be included in the averaging. For this work, a matrix of 5 by 5 (24 neighbors) was chosen. Other matrix sizes were tested, and 5 by 5 removed sufficient noise without oversmoothing.
- Powerline noise removal: The powerline noise at 60 Hz was removed from the recordings using an infinite impulse response band-stop filter.
- Temporal filtering: The signal of each pixel was temporally filtered using the Parks-McClellan Remez exchange algorithm, available as the MATLAB function "firpm", which applies a finite impulse response signal processing filter to the data. The low and high band-pass threshold frequencies chosen were 0 Hz and 100 Hz, respectively. The filter may cause a temporal shift, and so a zero-phase filter was also used (MATLAB function "filtfilt").
- Drift correction: A fourth-degree polynomial function was fitted to the signal of each pixel using "polyfit". The polynomial was then subtracted from the signal to remove any

existing drift.

Cycle length (CL) calculation

After filtering the data, the dominant frequency (DF) and the CL were extracted. The DF corresponds to the highest peak of the discrete fourier transform of the signal, that is, the frequency that carries the most energy. It is obtained for each pixel and therefore provides information about the cardiac rhythm and its stability across the myocardium. The CL is the time interval between activations. Because the relationship between frequency (f) and the period (T) of a sinusoidal signal is given by:

$$T = \frac{1}{f}$$

The inverse of DF already gives an idea of what the CL should be. However, a more accurate estimate of CL was attained by finding the activation peaks of the signal for each pixel and calculating the median of the difference between consecutive peaks. The MATLAB function “findpeaks” was used for this, and the median value of DF was used to determine target times to improve the peak detection. The CL was computed for each pixel. The lowest value of CL for each recording was considered the minimum CL.

Phase mapping

The conversion of fluorescence data to phase angle involved preparing the data and applying the Hilbert transform (Supplementary Figure S1). The first step of this operation is to subtract the mean from the signal because the phase calculation is sensitive to constant components. Let $f(t)$ be a filtered fluorescence signal: the zero-mean signal $\tilde{f}(t)$ is obtained through

$$\tilde{f}(t) = f(t) - \overline{f(t)},$$

$$z(t) = \tilde{f}(t) + iH\ \tilde{f}(t)$$

with $f(t)$ as the mean value of $f(t)$. Then, the signal is inverted, so that the phase cut matches with the cardiac depolarization. The Hilbert transform was performed using the MATLAB function "hilbert" from the Signal Processing Toolbox, which returns an analytical signal, $z(t)$, with the signal and its Hilbert transform as complex conjugates. In the phase-space, the real and the imaginary parts of $z(t)$ are plotted against each other and the phase angle, θ, can be computed for each instant. This relationship is described by

$$\theta(t) = \arctan \frac{imag\{z(t)\}}{real\{z(t)\}} = \arctan \frac{H[\tilde{f}(t)]}{\tilde{f}(t)}$$

The resulting phase data varies from -π to +π, corresponding to a complete cycle of the cardiac action potential.

The conversion of fluorescence data to phase adds noise to the data. Noisy phase maps are prone to phantom phase singularities (PSs) and make it harder to track the real ones. Therefore, spatial binning after phase mapping was added to the algorithm. The phase data were smoothed using the same method as for the fluorescence data spatial binning. However, to avoid noise in the transition line from $-\pi$ to $+\pi$, the phase angle data (θ) had to be converted to its exponential form ($e^{i\theta}$) before spatial smoothing and then reverted afterward. When applied directly, the smoothing operator does not respect the discrete transition between $-\pi$ and π (Supplementary Figure S2). This method avoids the erroneous transition. To verify if this step would be beneficial to track PSs and, consequently, a useful addition to the algorithm, the results of PS tracking with or without phase binning were compared for one recording. It was observed that smoothing the phase data facilitated tracking and prevented some PSs from being temporally broken into shorter duration ones (Supplementary Figure S3). Furthermore, it reduced the number of false rotor detections.

Downsampling

To test the effect of lower resolutions, the data were uniformly decimated by D downsampling factor, where $D \in \{2, 3, 4, 5, 6, 7, 8, 9, 10\}$. The pixels that were collected were considered pseudo-electrodes, as they mimic the information obtained from electrical

mapping. This was achieved by creating an interpolant based on the original data matrix through the MATLAB function "griddedInterpolant". The interpolant was then used to extract the values corresponding to the uniform grid for each sub-resolution.

As the default, the grid was positioned starting in the upper left corner of the original data matrix. However, to study the effect of the grid position on the results, three other placements were tested for D = $\{2, 5, 10\}$. These subresolutions were chosen to provide a range of high, intermediary, and low spatial resolutions. The downsampling was performed after filtering and phase mapping because optical data are very noisy and downsampling before its filtering would not provide realistic or reliable results. There was no impact of downsampling before or after phase mapping because the conversion was done on a pixel basis; therefore, that choice was arbitrary

Upsampling

The decimated data were upsampled back to the original resolution immediately after downsampling so that comparison with the original data would be possible. The MATLAB function "griddedInterpolant" was again used, in this instance to create an interpolant based on the sub-resolution data matrix. By using the interpolant, the values corresponding to the lost data points were computed through interpolation.

Three interpolation methods were tested to determine which provided the highest fidelity: bilinear, bicubic, and cubic spline. Bilinear and bicubic interpolations are extensions of linear and cubic interpolations for two-dimensional data. Bilinear interpolation connects the known data points with a straight line, bicubic interpolation uses a cubic convolution kernel, and cubic spline uses spline interpolation.

Data masking

Optical data are prone to noise and may not be spatially uniform. Therefore, to only analyze the meaningful and relevant areas of the recording, a data mask is created from a baseline recording and applied to the arrhythmic data. This step is performed after upsampling to avoid loss of information at the mask border.

PS (rotor) detection

An arrhythmic episode may be caused by an anatomic or functional anomaly in the tissue conductivity, inducing a reentrant pathway. When reentry occurs, a conducting wavefront re-excites part of the cardiac tissue it already passed through, pivoting around a core of unexcited but excitable cardiac tissue. This phenomenon can be recognized in phase maps as a phase singularity (PS), a point where the range of cardiac excitation values merges. Therefore, a PS is found by detecting sharp variation in the phase map, both

horizontally and vertically.

To identify those points, the partial derivative is calculated in both directions, resulting in two separate matrices for each time frame. The resulting values are adjusted to remain between -π and +π, replacing those higher than +π and lower than -π in the following manner:

$$\pi + a \Rightarrow -\pi + a \wedge -\pi - b \Rightarrow \pi - b, \text{ for } a, b > 0$$

The Sobel edge detection operator was convoluted with each matrix, its direction corresponding to the partial derivative direction. Both matrices were summed for each time frame, resulting in an overall variation description through time. Because both directions are considered, the variation of the isophase line when π transitions to -π is overlooked and only PSs, which represent a phase jump in every direction, are recognized.

The phase jumps were expected to be ±2π, and a tolerance of 3 rad was deemed appropriate. Consequently, values superior to 2π-3 rad were considered clockwise PS candidates, while those inferior to -2π+3 rad were considered anti-clockwise PS candidates (Supplementary Figure S4). Each PS is defined by four pixels in a square, that is, four neighboring PS candidates. To avoid mistakenly tracking the same PS more than once, the neighborhood rule was added to the algorithm.

When a PS candidate pixel is found, it verifies how many of its neighbors are PS candidates as well. If the pixel is inserted in a neighborhood of at least four pixels that are PS candidates and that have the same chirality, then the neighborhood is deemed a PS. If the

neighborhood fewer than four pixels, then it is considered noise.

After assessing that the neighborhood represents a true PS, the pixel with the highest variation in its neighborhood is appointed as the location of the PS. This step reduces computation time and makes the code more robust against considering PS candidates in the same neighborhood as different PSs (Supplementary Figure S5). Furthermore, ignoring isolated candidates avoids false detections on the border of the data mask.

PS tracking

To assess the lifespan and movements of each PS, it is necessary to track them through time and establish each individual path across the myocardium. To do so, a MATLAB object is created for every PS in every frame, with its location and chirality set as properties. For every frame in the recording, the PSs present are analyzed. If a PS is not already assigned to a path, its frame and index number are added to the PS object properties as the starting point of a new path. Then, its position is compared against the PSs in the next five frames. If a PS is found within a 2-pixel (1.4 mm) radius from the previous one and with matching chirality, it is deemed to be the same PS and added to the path. The choice of 1.4 mm for the allowed spatial displacement of the PS between frames is based on the maximum velocity of action potential propagation, which is 100 cm/s. Therefore, for a temporal displacement of 1 ms between frames, a PS's maximum movement should be 1 mm

due to physiological constraints. However, the spatial threshold should not be a fixed value but instead depend on the temporal displacement. For example, if the PS is only detected again after three frames because of the noise, the movement allowed should be 3 mm. The fixed value was implemented and thus constitutes a limitation to the current work.

For every PS added to a path, the path's lifespan, distance covered, and displacement between its initial and final positions are updated. PSs lasting less than 50 ms were considered noise and not included. The reasoning behind the temporal threshold of 50 ms was that some PSs were found in baseline recordings with lifespans up to 40 ms. Because these recordings represented sinus rhythm and, therefore, possessed no real PSs, it was confirmed that signal noise produced phantom rotors that could last that long. Consequently, as a precaution, all PSs detected in arrhythmic signals that lasted less than 50 ms were discarded as potential illusory rotors. For PS tracking, the CADENCE software was optimized by adding a PS blacklist. It was observed that on some occasions, one noisy PS point or PS segment could appear near another PS trajectory: due to proximity, the future trajectory for the other PSs could be attributed as its own. Consequently, some PSs would appear repeated (Supplementary Figure S6). To eliminate these cases, every instantaneous PS assigned to a trajectory was added to the PS blacklist and was not considered for future PSs being tracked.

PS density maps: peak detection

PS density maps provide an additional method to evaluate the detection of a PS and, more specifically, the localization accuracy, because these maps are used to visualize PS distribution. The density maps were created by counting a PS's occurrences at each pixel. For every PS lasting more than 50 ms, its path was registered by incrementing the PS count of the location where it appeared in each frame. To better compare the maps from different resolutions, and to better visualize PS distribution, the density maps were spatially averaged with a 3-by-3 pixels bin (Supplementary Figure S7).

To detect the density peaks, the MATLAB function "imregionalmax" was used. This function finds the regional maxima in each image. This method, however, led to the detection of not only the true peaks but also of any slight peaks in low-density regions. To isolate the true peaks, some conditions had to be implemented. First, a minimum density was defined. To do this, the pixel with the highest PS density was detected; for any other possible peaks to be considered so, their PS density had to be at least 75% of the value of highest density. This step cut the false low-density peak detections. The second condition was aimed at avoiding multiple detections of the same peak. For any potential PS density peak, the pixels within a certain radius were also considered, to determine if there were other possible peaks nearby. If so, the highest peak was kept and all other peaks in the neighborhood were discarded (Supplementary Figure S8).

Correlation between original and decimated data

To analyze how a decrease in spatial resolution affects the dynamics of arrhythmia drivers, it is necessary to determine if the PSs found at the original resolution are also present in the decimated maps and, if so, how they differ. When analyzing density maps, it is also necessary to appraise the corresponding peaks in the original maps and in the subresolution maps. This section describes how these correlations were established for both the individual PSs and the PS density map's peaks, and explains which information was retrieved from them.

Which, if any, of the PSs correlated spatially and temporally with the ones from the original maps was determined for each subresolution. When comparing the PSs present in the original map and the decimated map, all possible pairings were analyzed one by one. Four conditions had to be fulfilled for a correlation to be drawn:

Condition 1. *Lifespan longer than the minimum CL.*

Only stable rotors were considered for correlation. A stable rotor (SR) was defined as lasting longer than the minimum CL of the recording. Note that one of the donor hearts studied (identified as D3 in Supplementary Table 1) had no stable rotors.

Condition 2. *Same chirality.*

PSs must rotate in the same direction to be considered equivalent.

Condition 3. *Share at least one-quarter of the lifespan.*

By changing the resolution of the data, the signal will be distorted and, in some instances, the flow of a rotor's path might be broken. The path is still present but separated into shorter-lasting fragments. If lasting longer than the minimum CL, the temporal restrictions of the fragments must be balanced to be considered a match to the original path. Although the rotors do not have to appear and disappear at the same time or have a similar lifespan, they must temporally correlate for at least one-quarter of their duration.

<u>Condition 4.</u> *Minimum spatial displacement of 5 pixels.*

The distance between the PSs' locations was measured for each shared frame. To be considered the same rotor, the mean distance between the locations had to be, ≤5 pixels, which corresponds to 3.5 mm. This value coincides with the mean radius of a tissue ablation lesion and therefore was considered an acceptable margin of error. This distance was taken as default, but other values were also evaluated.

The correlation process was divided into three steps. First, the primary matches were established, where each rotor was only allowed to match once. Thus, if there were fragmented drivers for any of the resolutions, the matching rotor would only correlate with one of the fragments. Rotors were classified into three groups: successfully matched rotors (those with the established pairings), missed rotors (uncorrelated PSs from the full resolution data), and false rotors (uncorrelated PSs from the subresolution data).

The second step was to check for correlations between the missing rotors and the

sub-resolution PSs. The objective was to confirm if the rotors were in fact missing or if multiple PSs from the original data represented a single PS from the decimated data. If any such pairing was found, the PS would be reclassified from the "missing rotors" bin to a new division, the mistakenly missed rotors.

Lastly, the same logic was applied to the false rotors: the mistakenly false rotors were separated from the false ones. In the end, the number of found rotors, which represents all the stable rotors from the original resolution that were identified in the sub-resolution maps, was given by the sum of the initially matched rotors and the falsely missed rotors.

In the case of PS density maps peaks, the position of each peak in the original maps was compared against the position of every peak at each sub-resolution. Peaks in the decimated maps were considered correlated if they were within a radius *r* from a true peak. To test the correlation success for different spatial accuracy requirements, multiple *r* values were explored (1.4 mm, 2.1 mm, and 3.5 mm).

The number of found peaks was the number of original peaks found in a sub-resolution PS density map, the number of missed peaks was the number of original peaks not found, and the number of false peaks was the quantity of erroneous peaks found in the sub-resolution map that were not present in the original.

Validation with electrical data

The optical maps were compared with the data from the electrodes array, to confirm correlation of the signals. To do so, the electrical signals had to be processed and converted to phase. This data processing was different from the processing of optical data. Not all electrodes recorded good-quality signals; noisy recordings were discarded (Supplementary Figure S9). Then, the data were filtered, and the derivative was used to find the activation temporal sites. After phase mapping, the data were interpolated to match the corresponding area in the optical maps.

Electrical signals are different from fluorescence signals, and therefore need a separate filtering process, that goes as follows.

- *Temporal filtering:* Each electrical signal was band-pass filtered using a high pass threshold of 2 Hz and a low-pass threshold of 40 Hz. These filtering settings removed high-frequency noise (Supplementary Figure S10) and provided a slight drift correction, thus effectively cleaning the action potential signals. The Parks-McClellan Remez exchange algorithm was used for this step, as described for the optical data temporal filtering.
- *Derivative:* To detect the depolarizations, the derivative of the signals was extracted.
- *Squaring:* To enhance the signal's peaks, which correspond to the depolarizations, each signal was squared.
- *Invert data:* For the phase transitions to match the signals' depolarizations, the signals

were inverted.

- *Smoothing:* A moving average was applied to the signals, with a moving window of 50 frames. The objective was to bring the signal as close as possible to a sinusoidal shape, and therefore resemble more accurately a fluorescence signal, so that the shape of the phase signals was similar and comparable. The averaging was done twice to achieve the desired shape. The conversion to phase was done as explained for the optical data.

Just as sub-resolution optical maps were upsampled back to the original resolution to enable comparison between the different maps, electrical data were similarly upsampled. To precisely compare the matching electrical and fluorescence signals, the positions of the electrodes in the optical mapping field of view were identified to create an electrode mask (Supplementary Figure S11). This mask with the electrodes' positions was used to interpolate the electrical data, using the MATLAB function "scatterInterpolant", and to downsample the optical data to 64 signals, as obtained for the electrical data. The temporal signals and the phase maps for the electrical and the optical data were visually compared.

Arrhythmia Dynamics Metrics

Incidence

Phase singularity incidence refers to the number of PSs present in each recording, and it was

measured as the total number of PSs found in the 8-s recording divided by the data mask area. The number of PSs is informative regarding the stability of the arrhythmia, as more chaotic rhythms have a higher incidence of PSs.

Duration

The duration, or lifespan, of a PS is the time interval from the instant the PS appears to when it disappears. For therapy purposes, the longer-lasting rotors are the ones considered relevant. Therefore, a substantial variation in this value would have an impact, as important information may be hidden or lost. This metric was calculated for all PSs and for stable rotors only. If no rotors were detected for any resolution of a given recording, the duration value was omitted.

Displacement

Displacement is given by the two-dimensional Euclidean distance between each PS found in a sub-resolution map and its original counterpart if a successful correlation was made. The Euclidean distance measures the length of a line segment connecting two points. For two dimensions, it is given by

$$d(p,q) = \sqrt{(p_x - q_x)^2 + (p_y - q_y)^2},$$

where p_x and q_x being the horizontal coordinates of points p and q, and p_y and q_y the vertical coordinates. At each sub-resolution of each recording, the mean and maximum displacement were extracted. For the cases where no rotor was found in the original optical map, there was effectively no displacement to be measured, and so the value was omitted.

F1-score for rotor detection

To verify a correlation success at each sub-resolution, three measures were considered: the number of true rotors found, the number of rotors missed, and the number of false rotors. These can be described in the following manner:

- *True positive:* True rotor found at a sub-resolution.
- *False positive:* False rotor found at a sub-resolution.
- *False negative:* True rotor not found at a sub-resolution.

This definition makes it possible to apply algorithm performance metrics, such as precision and recall (also known as sensitivity). Precision is the ratio of the true rotors found to all the rotors found at a sub-resolution, whereas recall is the ratio of the true rotors found at a sub-resolution to all the rotors found in the original resolution.

$$\text{Precision} = \frac{\text{True positives}}{\text{True positives} + \text{False positives}} = \frac{\text{Rotors found}}{\text{Rotors found} + \text{False rotors}}$$

$$\text{Recall} = \frac{\text{True positives}}{\text{True positives} + \text{False negatives}} = \frac{\text{Rotors found}}{\text{Rotors found} + \text{Missed rotors}}$$

Precision and recall can be summarized by the F1-score, which is the harmonic mean of the two metrics

$$\text{F1-score} = 2 \times \frac{\text{Precision} \times \text{Recall}}{\text{Precision} + \text{Recall}}$$

The F1-score has been used to evaluate PS detection algorithms and was chosen to evaluate correlation performance in this work. It ranges from 0 to 1: 0 if no correlation is made, and 1 if all the original rotors are found and no different rotors are detected. For the cases where no rotor was found in the original optical map, the F1-score was omitted.

F1-score for PS density map peaks

The peaks of the PS density maps were used to assess if the locations with highest PS incidence were the same across resolutions. To compare the PS density peaks at multiple sub-resolutions regarding the original, the F1-score was used again. In this instance, true positives were the correctly found peaks, false positives were the erroneous density peaks, and false negatives were the undetected density peaks

Categorial analysis

The rotor data extracted from the optical recordings were further analyzed according to

following inherent characteristics.

Arrhythmia type

MVT and VF recordings were analyzed separately to assess type-specific arrhythmia dynamics and rotor detection accuracy.

Sex

Sex differences were analyzed by discriminating the recordings obtained from female and male donors.

Anatomical region

To verify and take into account possible regional differences in arrhythmia dynamics, the field of view was divided into three sections: the RVOT region, the mid- ventricular (MID) region, and the apical (API) region (Supplementary Figure 12). The area of the data mask in each region was considered for analysis.

Mapped surface

For every experiment, the endocardium and the epicardium were mapped simultaneously.

The results obtained for each surface were compared.

Statistical analysis

The SEM rather than SD was used for trends across the cohort of donor hearts because it measures how far the sample mean is from the true population mean. When studying accuracy profiles, the 95% confidence interval of the mean was also presented. All datasets were assumed to have a normal distribution, because the metrics analyzed were derived from normal data. Incidence, duration, and displacement metrics were displayed as box and whiskers plots, with the box limits marking the quartiles and the whiskers marking the minimum and maximum values.

For repeated measures ANOVA tests, sphericity was not assumed, and corrections were made using the Greenhouse-Geisser method. Variance homogeneity was verified through the Brown-Forsythe test and Bartlett's test. When relevant, post hoc multiple comparisons tests were performed.

Supplementary Table S1. Donor human heart information

Donor #	Age	Sex	Race	COD	LVEF	BMI	Drugs	Alcohol	Smoker	DM	HTN	Heart Disease
D1	62	M	Black	Brain Death	NA	23.3	Yes	Yes	Yes	No	No	No
D2	65	F	White	Stroke	NA	36.3	No	Yes	Yes	No	Yes	No
D3	71	F	White	Stroke	65%	26.6	No	Yes	No	No	No	No
D4	63	F	White	Stroke	NA	26.9	No	No	No	No	No	Sinus Tac
D5	49	M	Hispanic	Head T	70%	36.3	No	Yes	Yes	No	No	Mild LVH
D6	57	M	Black	Stroke	NA	40.2	No	Yes	No	No	Yes	No
D7	57	F	Black	Stroke	70%	29.2	No	Yes	Yes	No	No	No
D8	62	M	Hispanic	Stroke	NA	18.6	No	Yes	Yes	Yes	No	No
D9	68	F	Black	Brain Death	60%	30.4	No	No	No	No	Yes	Mild LVH
D10	60	F	White	Brain Death	NA	20.8	No	Yes	Yes	No	No	Sinus Tac
D11	56	M	White	Brain Death	55%	26.9	Yes	No	Yes	Yes	No	Sinus Tac
D12	51	M	White	Stroke	NA	21.5	Yes	Yes	Yes	No	No	MI

COD = cause of death, LVEF = left ventricular ejection fraction, BMI = body mass index, DM = diabetes mellitus, HTN = hypertension.

Heart disease includes sinus tachycardia, left ventricular hypertrophy, coronary arterial disease, and myocardial infarction

Supplementary Table S2. Summary of experiments

Donor ID	D1	D2	D3	D4	D5	D6	D7	D8	D9	D10	D11	D12
n	3	2	2	2	2	2	2	2	2	2	2	2
Sex	M	F	F	F	M	M	F	M	F	F	M	M
A-Type	MVT	VF	VF	MVT	MVT	VF	VF	VF	VF	MVT	MVT	VF

For each experiment, both sides of the heart were recorded simultaneously, and so the number of recordings per heart is 2n.

n = number of experiments for each heart, M = male, F = female, A-type = arrhythmia type, MVT = monomorphic ventricular tachycardia, VF = ventricular fibrillation

Supplementary Table S3. Comparative table of currently used clinical mapping catheters.

The mean IED is ~4 mm

**Catheter**	**Style**	**NE**	**D (mm)**	**IED (mm)**
Constellation™[1]	Basket	64	31 to 75	2 to 7
IntellaMap Orion™[1]	Basket	64	22	2.5
Advisor™ HD Grid[2]	Grid	16	N/A	3
Inquiry™ Afocus II™[2]	Double Loop	20	N/A	4
Lasso® Nav[3]	Single Loop	10 or 20	N/A	8 or 2-6-2
Pentaray® Nav[3]	Star	20	N/A	4-4-4 or 2-6-2
Decanav®[3]	Linear	10	N/A	2-8-2

NE = number of electrodes; D = diameter, IED = inter-electrode distance

[1]Boston Scientific, Marlborough, Massachusetts, USA

[2]Abbott, Plymouth, Minnesota, USA

[3]Biosense-Webster, Irvine, California, USA

Supplementary Figures

Supplementary Figure S1

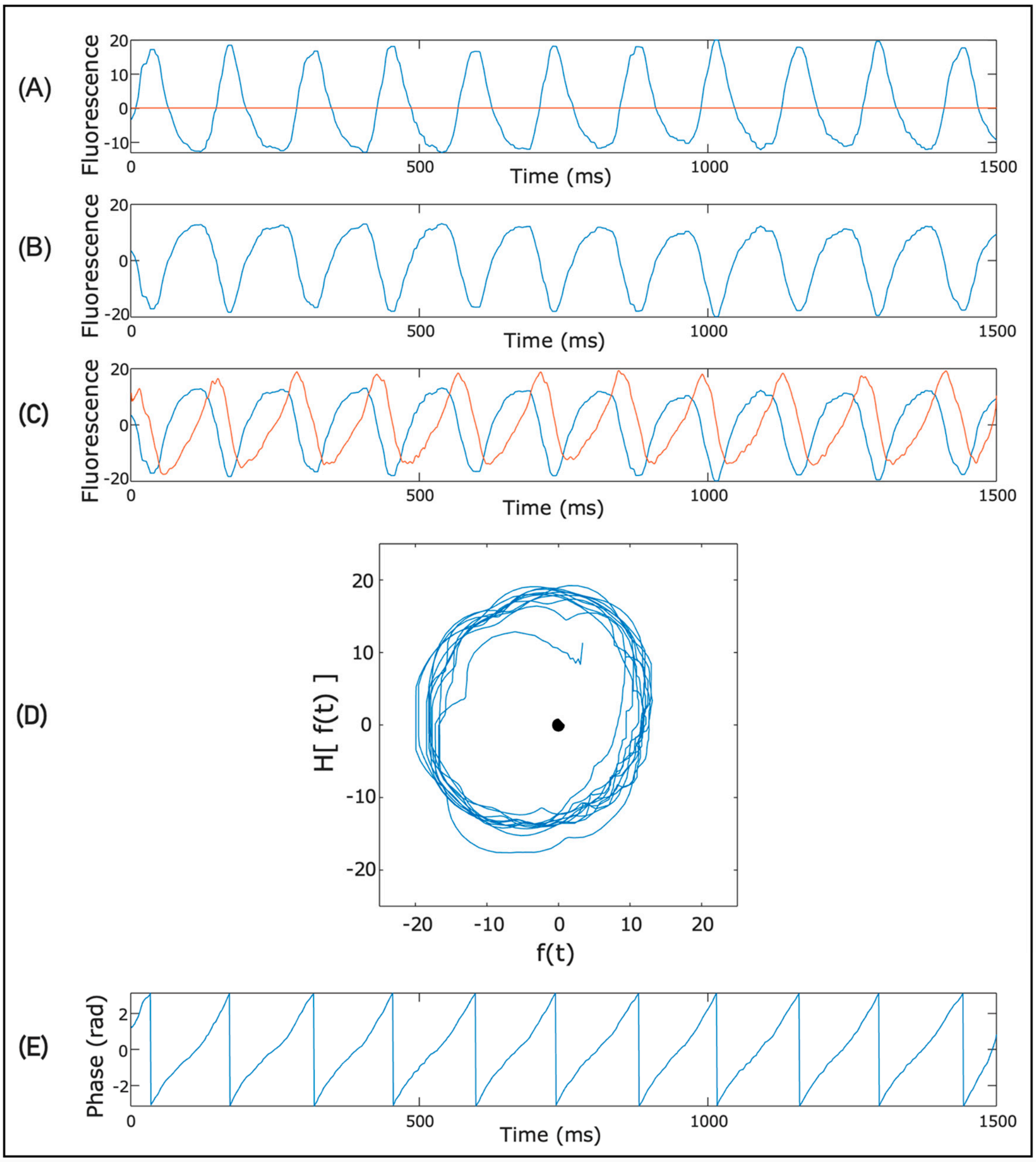

Supplementary Figure S1. Phase mapping process

(A) The filtered signal (blue) and the mean of the signal (orange), which in this case is zero. Therefore, the signal remains the same after subtracting the mean. (B) The signal is inverted so that the phase cut matches the signal's peaks. (C) Inverted signal (blue) and its Hilbert transform function (orange), with a phase-shift of 90º. (D) Inverted function is plotted against its Hilbert transform. (E) Phase angle from the graph in panel D, plotted in time. The transition from π to -π corresponds to the filtered signal's peaks, and each segment from -π to π corresponds to one cardiac cycle.

Supplementary Figure S2

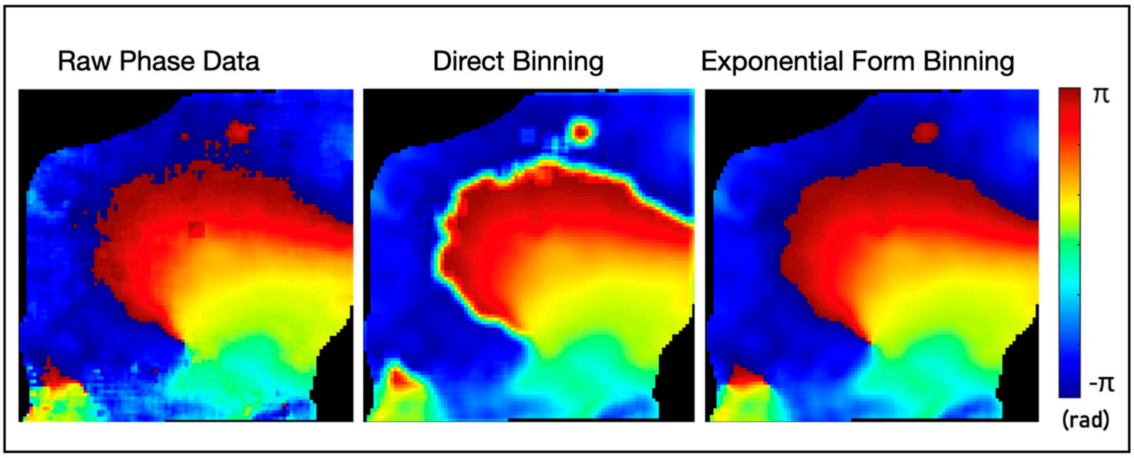

Supplementary Figure S2. Spatial binning

Spatial binning was applied after converting phase angle to exponential form. Phase data binning was performed to reduce noise. The data had to be converted to an exponential form before spatial averaging to avoid issues in the transition from π to -π. After smoothing, the

data were returned to phase angle. In this case, the bin size of the smoothing operator was 5 by 5 pixels.

Supplementary Figure S3

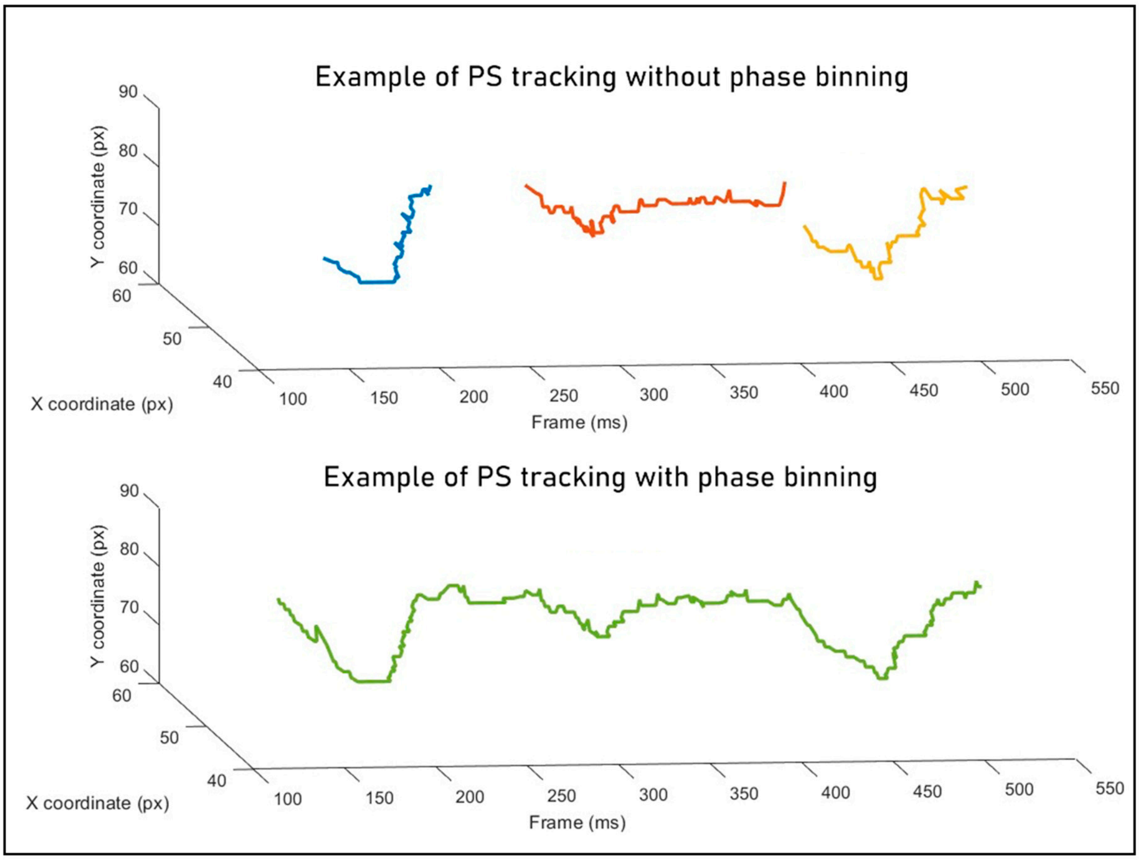

Supplementary Figure S3. Comparison of PS tracking with or without phase binning.

Each line segment in the graphs corresponds to a phase singularity (PS), represented by their spatial coordinates through time. The three rotors detected without phase binning for this temporal segment (top) correlate with a single rotor detected when spatially averaging the phase data (bottom).

Supplementary Figure S4

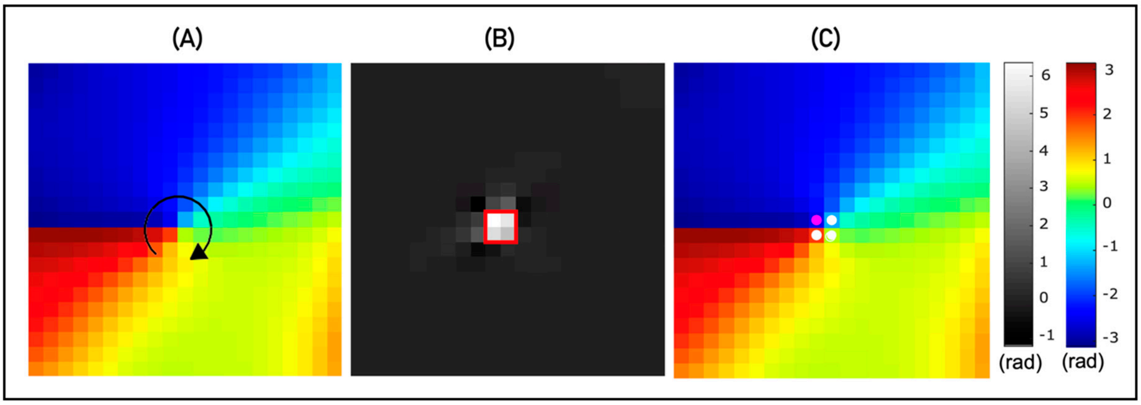

Supplementary Figure S4. PS detection process

(A) Section of one instantaneous phase map where one clockwise phase singularity is present. (B) Output of the convolution of the partial derivatives of the phase map with the Sobel operator. The red square delimits a neighborhood of four pixels that have a phase variation high enough to be considered phase singularity (PS) candidates. Because the neighborhood has at least four members, it is not discarded as noise. The neighbor with the highest absolute value is selected to represent the PS. (C) Phase map with the PS candidate's neighborhood marked by the dots. The pink dot signals the selected candidate, and the remainder are in white.

Supplementary Figure S5

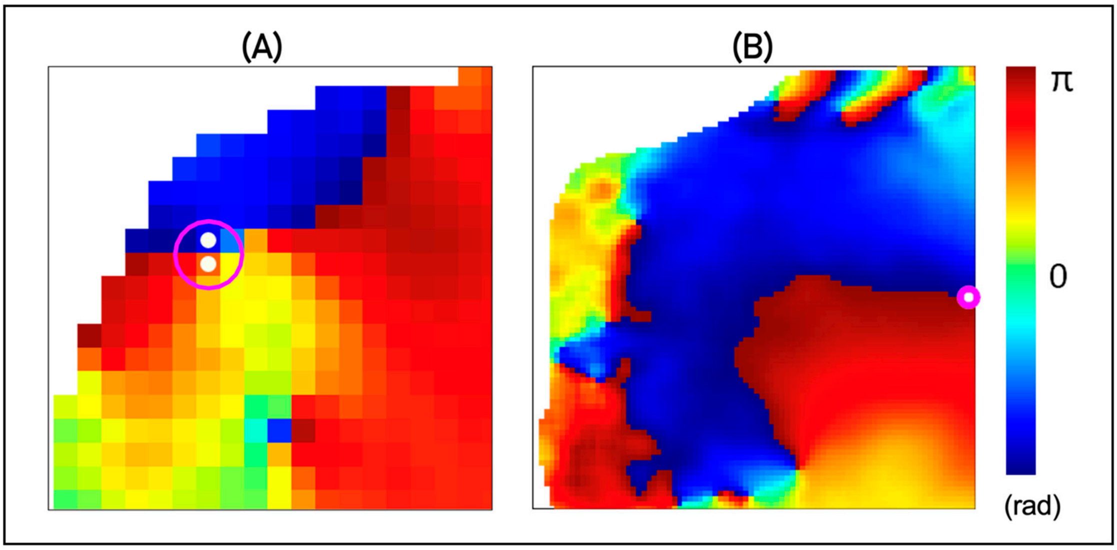

Supplementary Figure S5. Examples of wrong PS detections that can be avoided with the neighborhood rule

(A) Double detection of the same phase singularity (PS). The white dots identify the PSs detected and are emphasized by the magenta circle. (B) False PS due to the image border, marked with a magenta circle.

Supplementary Figure S6

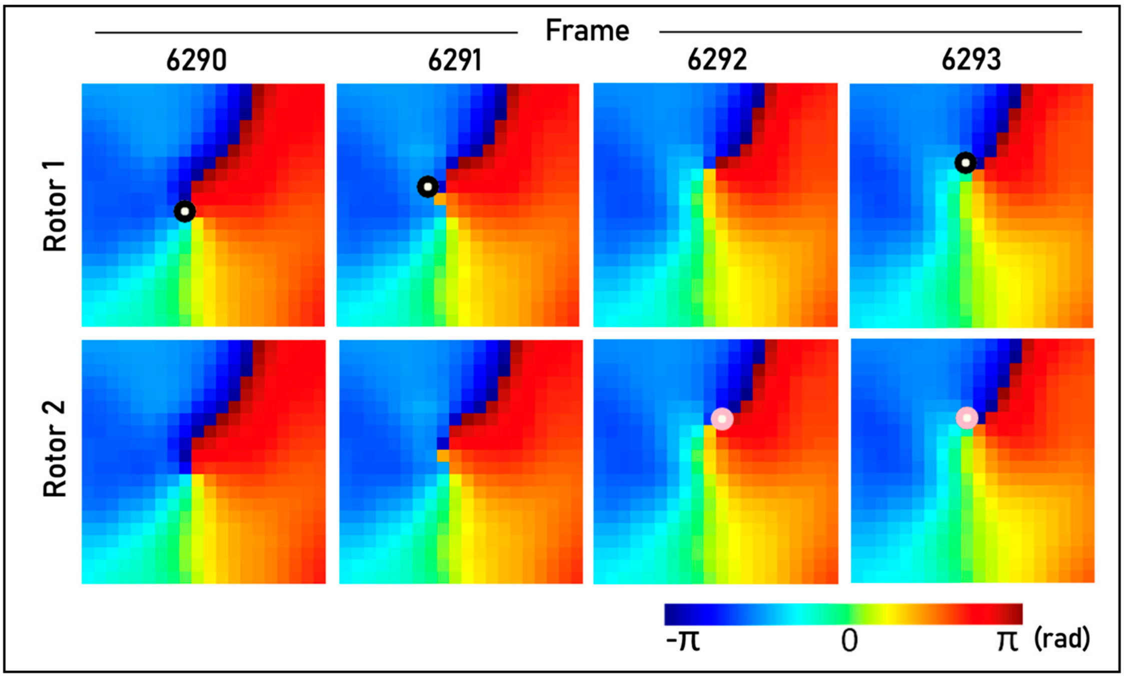

Supplementary Figure S6. Rotor overlap without PS blacklist

Four-second segment of two separate phase singularity (PS) trajectories detected, Rotor 1 and Rotor 2. Rotor 1 starts in frame 6269, whereas Rotor 2 appears in frame 6292 displayed. Rotor 1 is lost in frame 6292 due to the spatial distance between instantaneous PSs but is reconnected in frame 6293. However, because the PS did not disappear in frame 6292, it was simply too distant, there is an instantaneous PS that is detected and used to start a new trajectory. From frame 6293 forward, the paths of Rotor 1 and Rotor 2 are the same. Thus, Rotor 2 is a duplication of a portion of Rotor 1. To prevent duplicate PSs, the PS blacklist is applied and for the presented case, Rotor 2 is not detected anymore.

Supplementary Figure S7

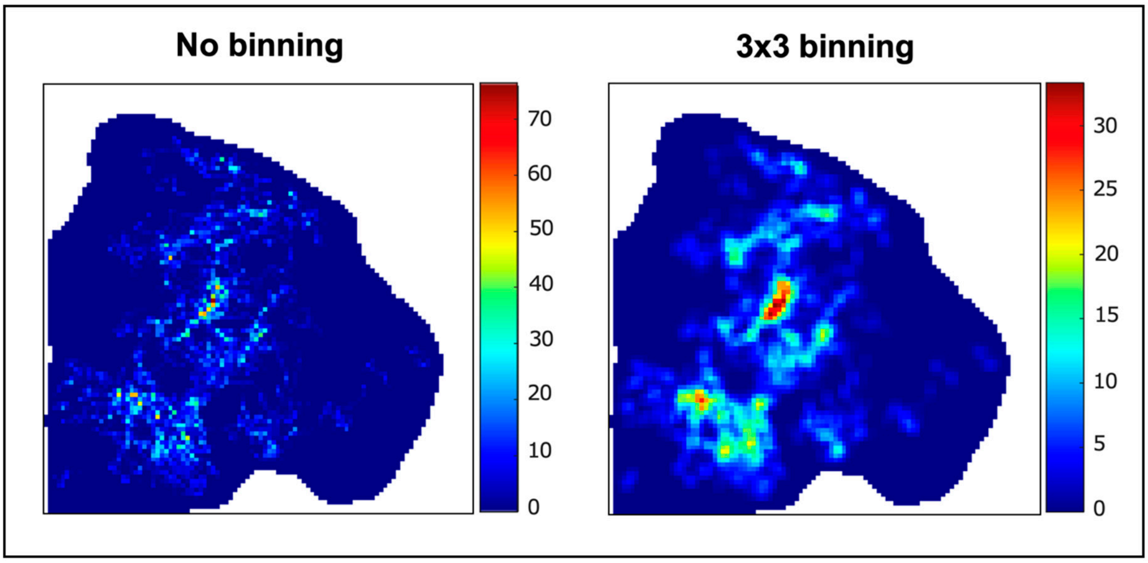

Supplementary Figure S7. PS density maps with and without spatial averaging

Because phase singularities (PSs) are not expected to be static, the averaged density map gives a more realistic notion of where PSs are present and where they are concentrated. A 3-by-3 pixels bin was used for the convolution.

Supplementary Figure S8

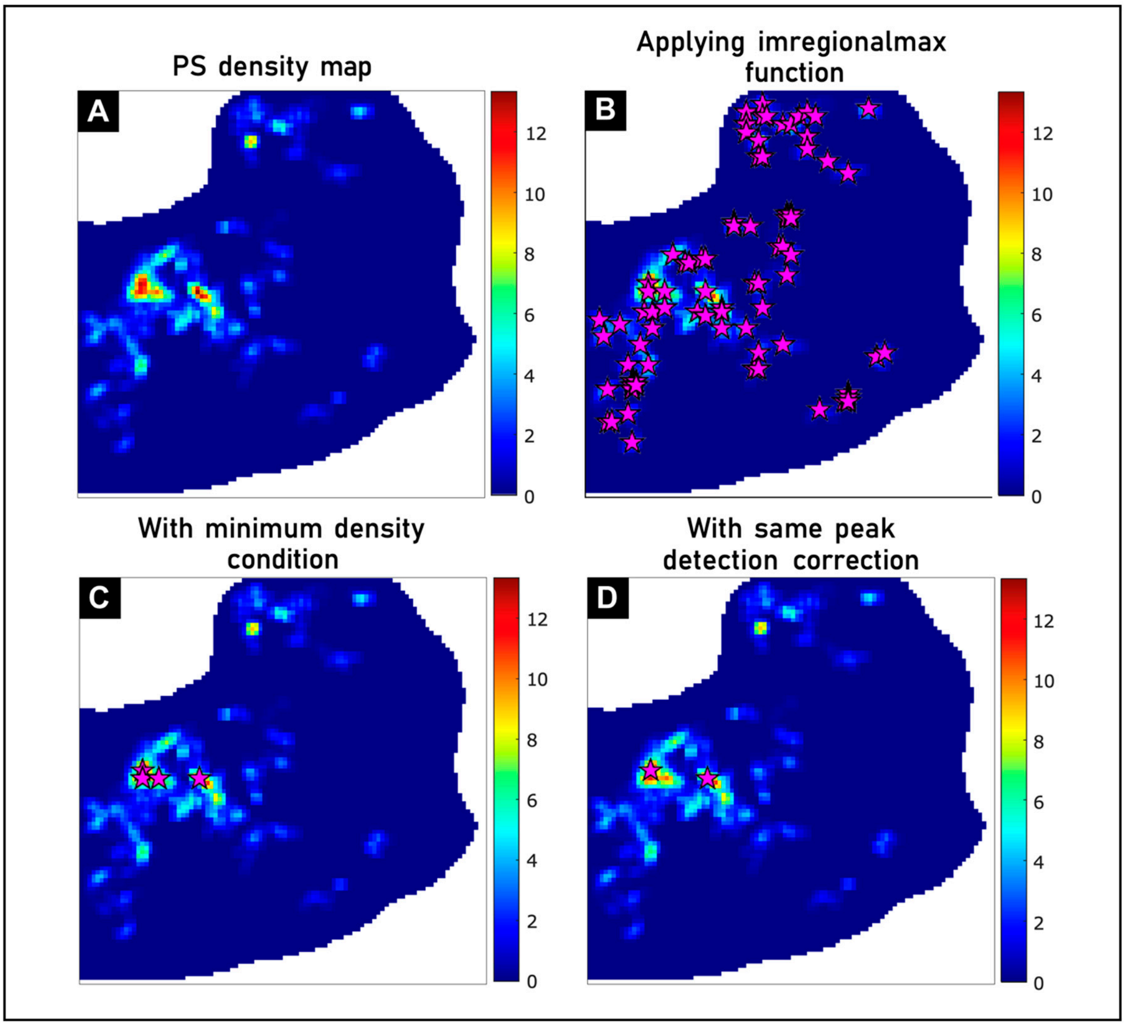

Supplementary Figure S8. Detection and conditioning of PS density peaks

Peak detections are represented by pink stars. (A) Representative PS density map. (B) Peak detection through MATLAB function "imregionalmax". Every deflection is considered a peak. (C) Peak detection after applying the minimum density conditioning. Only peaks with density ≥75% of the highest peak are considered. (D) Final density peaks after correcting multiple detections of the same peak. Only the highest peak within a certain radius is

considered a true peak.

Supplementary Figure S9

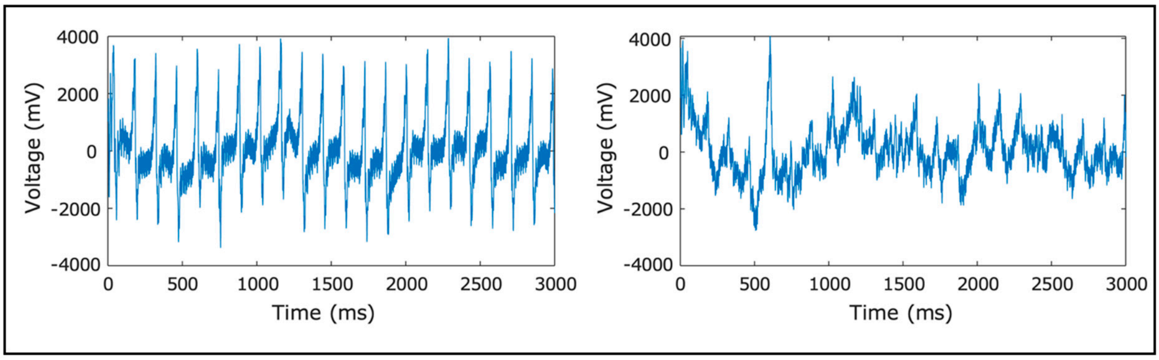

Supplementary Figure S9. Representative signals of viable and non-viable electrical raw data

The viable signal, on the left, has clear activations, whereas the non-viable one (right) has no distinguishable activation points and no organization.

Supplementary Figure S10

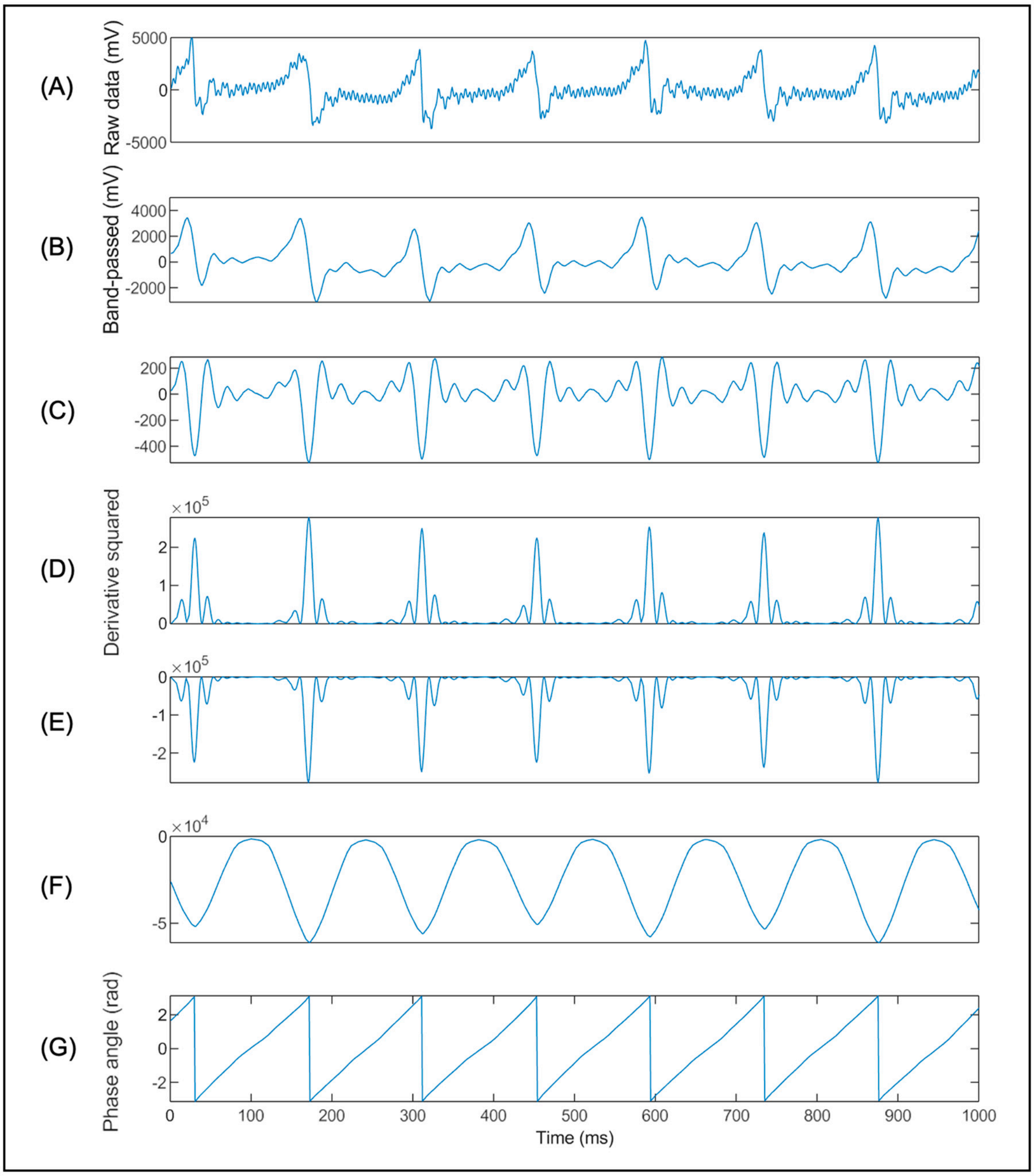

Supplementary Figure S10. Steps for filtering electrical data and converting it to phase

(A) Raw electrical data, which were preprocessed at the time of acquisition with a band-pass filter of 0-200 Hz and notch filtering. (B) Data after frequency filtering with a

band-pass of 2-40 Hz. (C) Approximate derivative of the signal. (D) The derivative is squared to enhance the peaks. (E) Signal is inverted so that the phase branch cut matches the depolarization. (F) The signal is smoothed twice with a moving average window of 50 frames, to resemble a sinusoidal wave and provide a cleaner phase signal. (G) Phase angle signal.

Supplementary Figure S11

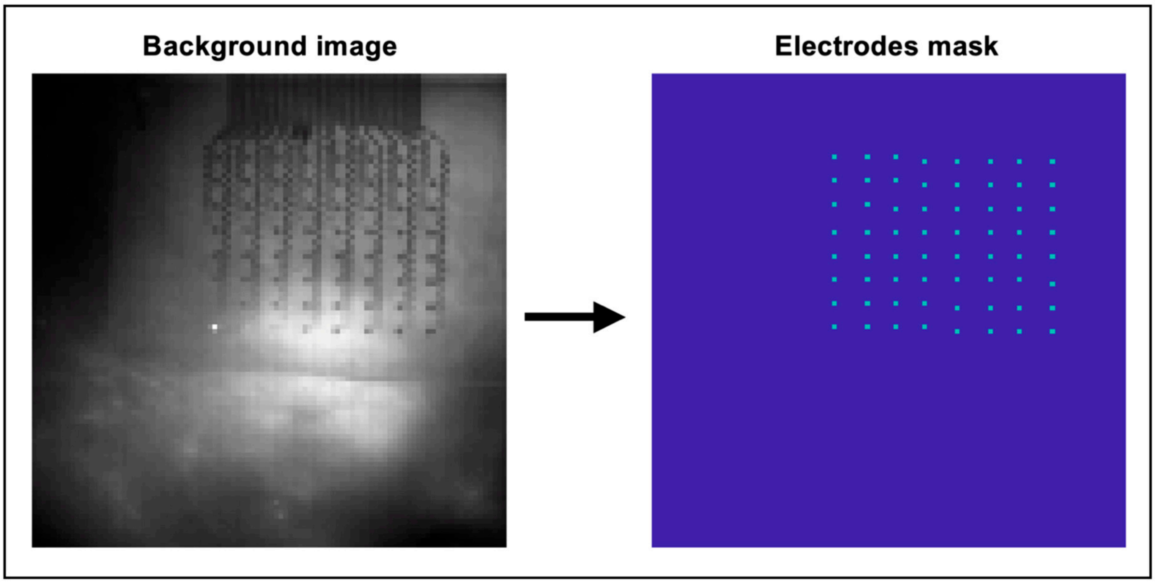

Supplementary Figure S11. Electrodes mask obtained from the position of the electrodes visible in the background images from the optical recordings.

This mask was used as a reference for both the downsampling of the optical data and the interpolation of the electrical data.

Supplementary Figure S12

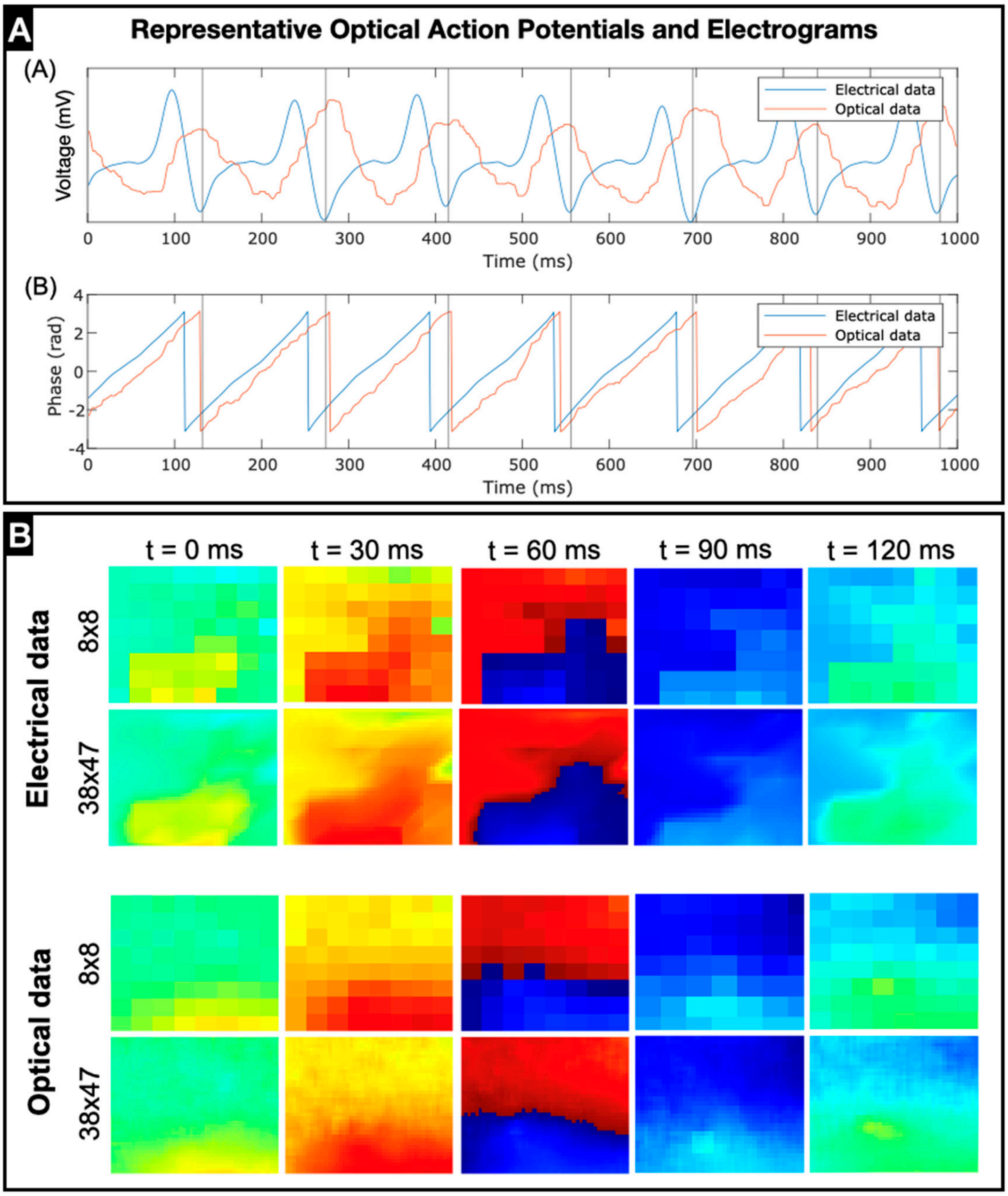

Supplementary Figure S12. Correlation between electrical and optical signals

(A) Representative 1- s segments of electrical and optical data recorded simultaneously

(Subpanel A). The vertical black lines mark the peaks of depolarization of the electrical signal (blue line). The fluorescence excitation intensity increases when membrane potential decreases; therefore the positive peaks for the fluorescence signal (orange line) are expected to be synchronized with electrical depolarization, which stands true for the segment presented. Both signals were normalized for comparison. (Subpanel B) Phase data from the signals in pane A. For optical data, the phase cut corresponds to the fluorescence peak and, therefore, to the end of the depolarization in the electrical data. For electrical data, however, the cut transition occurs in the middle of the depolarization, which explains the slight delay of the optical phase signal in relation to the electrical. (B) Correlations between electrical and optical phase maps.

Supplementary Figure S13

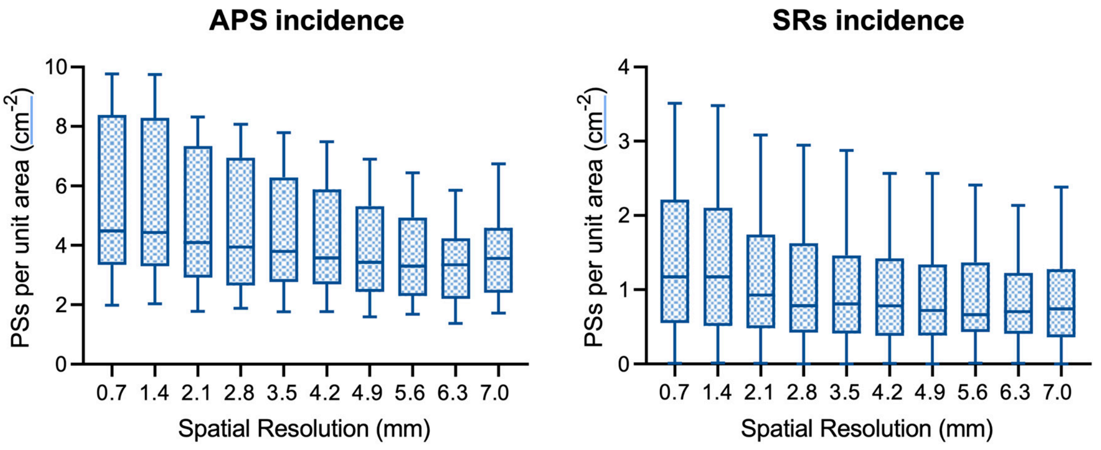

(a) All phase singularities (N=12).

Comparison	Summary	*p* value
0.7 vs. 1.4	ns	0.4007
0.7 vs. 2.1	**	0.0029
0.7 vs. 2.8	**	0.0078
0.7 vs. 3.5	**	0.0091
0.7 vs. 4.2	**	0.0095
0.7 vs. 4.9	**	0.0059
0.7 vs. 5.6	**	0.0090
0.7 vs. 6.3	**	0.0066
0.7 vs. 7.0	**	0.0095

ns, not significant.
** p value ≤ 0.01.

(b) Stable rotors (N=11).

Comparison	Summary	*p* value
0.7 vs. 1.4	ns	0.1424
0.7 vs. 2.1	**	0.0063
0.7 vs. 2.8	**	0.0072
0.7 vs. 3.5	*	0.0136
0.7 vs. 4.2	*	0.0152
0.7 vs. 4.9	*	0.0123
0.7 vs. 5.6	*	0.0221
0.7 vs. 6.3	*	0.0338
0.7 vs. 7.0	*	0.0437

ns, not significant.
* p value ≤ 0.05; ** p value ≤ 0.01.

Supplementary Figure S13. Rotor incidence

(Top) Incidences of all phase singularities (APS; includes stable as well as unstable rotors) and stable rotors (SRs) at different spatial resolutions (n = 12). (Bottom) Results of Dunnett's multiple-comparisons test to assess significant differences in rotor incidence due to spatial

resolution. The mean at each subresolution was compared to the control, which is the original resolution's mean rotor incidence. The test was performed for the incidence of APS (a) and incidence of SRs (b).

Supplementary Figure S14

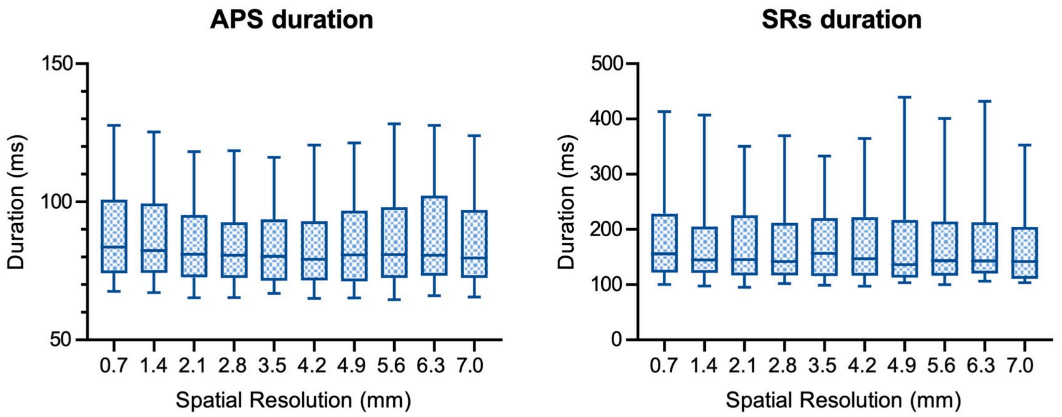

(a) All phase singularities (N=12).

Comparison	Summary	*p* value
0.7 vs. 1.4	ns	0.0828
0.7 vs. 2.1	**	0.0037
0.7 vs. 2.8	**	0.0014
0.7 vs. 3.5	**	0.0058
0.7 vs. 4.2	**	0.0015
0.7 vs. 4.9	*	0.0108
0.7 vs. 5.6	*	0.0152
0.7 vs. 6.3	ns	0.9999
0.7 vs. 7.0	ns	0.5609

ns, not significant.
* p value ≤ 0.05; ** p value ≤ 0.01.

(b) Stable rotors (N=11).

Comparison	Summary	*p* value
0.7 vs. 1.4	ns	0.3554
0.7 vs. 2.1	ns	0.2068
0.7 vs. 2.8	ns	0.0991
0.7 vs. 3.5	ns	0.4005
0.7 vs. 4.2	ns	0.0974
0.7 vs. 4.9	ns	0.9997
0.7 vs. 5.6	*	0.0450
0.7 vs. 6.3	ns	0.9888
0.7 vs. 7.0	ns	0.2585

ns, not significant.
* p value ≤ 0.05.

Supplementary Figure S14. Rotor duration

(Top) Durations of all phase singularities (APS; includes stable and unstable rotors) and stable rotors (SRs) at different spatial resolutions (n = 11). (Bottom) Results of Dunnett's multiple comparisons test to assess significant differences in rotor duration due to spatial resolution. The test was performed for the duration of APS (a), and duration of SRs (b).

Supplementary Figure S15

Resolution (mm)	F1-score	Confidence Interval
1.4	0.85 ± 0.02	0.80 - 0.90
2.1	0.70 ± 0.04	0.60 - 0.80
2.8	0.70 ± 0.03	0.62 - 0.78
3.5	0.64 ± 0.04	0.56 - 0.71
4.2	0.57 ± 0.04	0.47 - 0.67
4.9	0.53 ± 0.04	0.43 - 0.63
5.6	0.53 ± 0.04	0.43 - 0.63
6.3	0.51 ± 0.05	0.41 - 0.62
7.0	0.42 ± 0.06	0.29 - 0.55

Supplementary Figure S15. Stable rotors detection accuracy

F1-scores for rotor detection accuracy and respective confidence interval for each spatial resolution for all hearts (n = 11). F1-scores are presented as means±SEMs.

Supplementary Figure S16

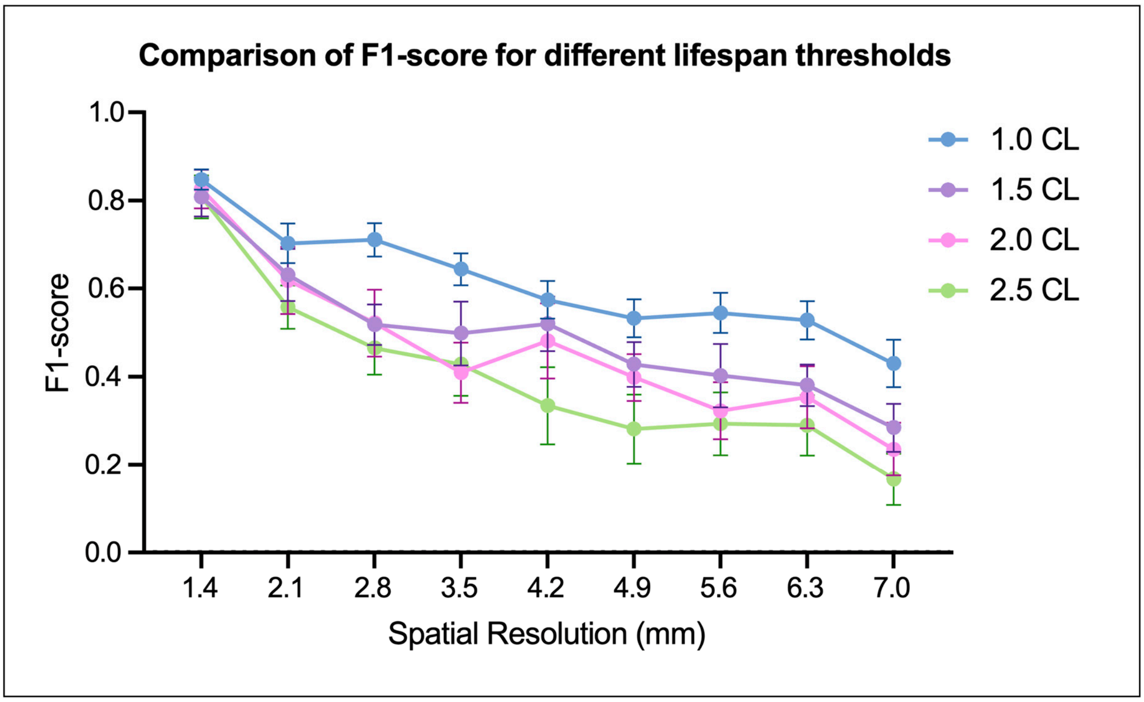

Supplementary Figure S16. F1-score of increasing PS duration

Comparison of F1-scores for different PS lifespan thresholds (n=11). F1-scores presented as means±SEMs, CL = cycle length.

Supplementary Figure S17

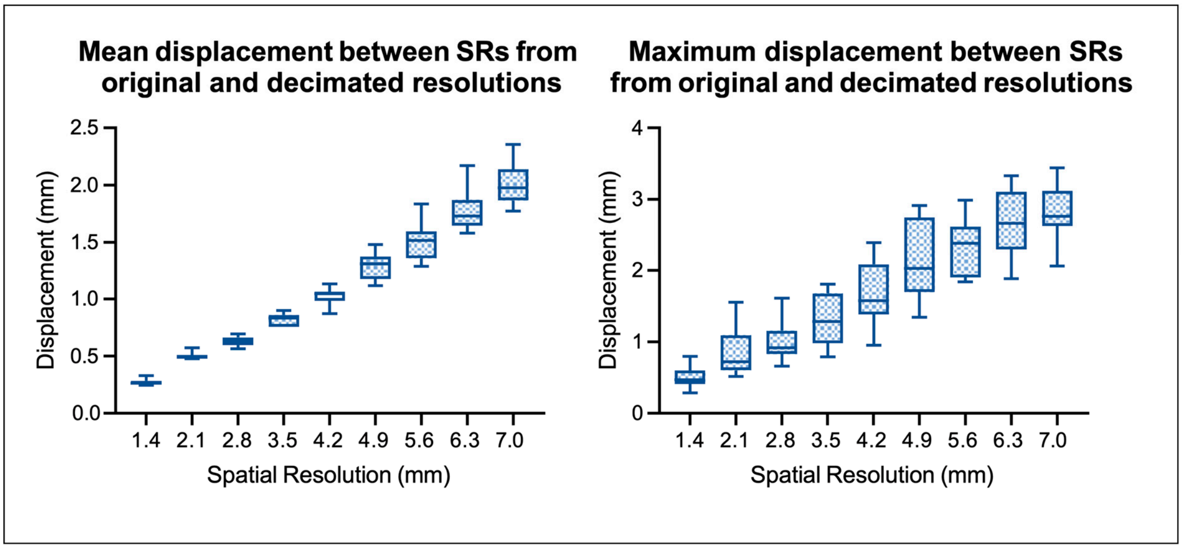

Supplementary Figure S17. Location accuracy

Mean and maximum displacement between successfully correlated rotors at different spatial resolutions (n = 11). Note that these values are constrained by the default displacement set for the correlation algorithm (spatial accuracy threshold = 3.5mm)

Supplementary Figure S18

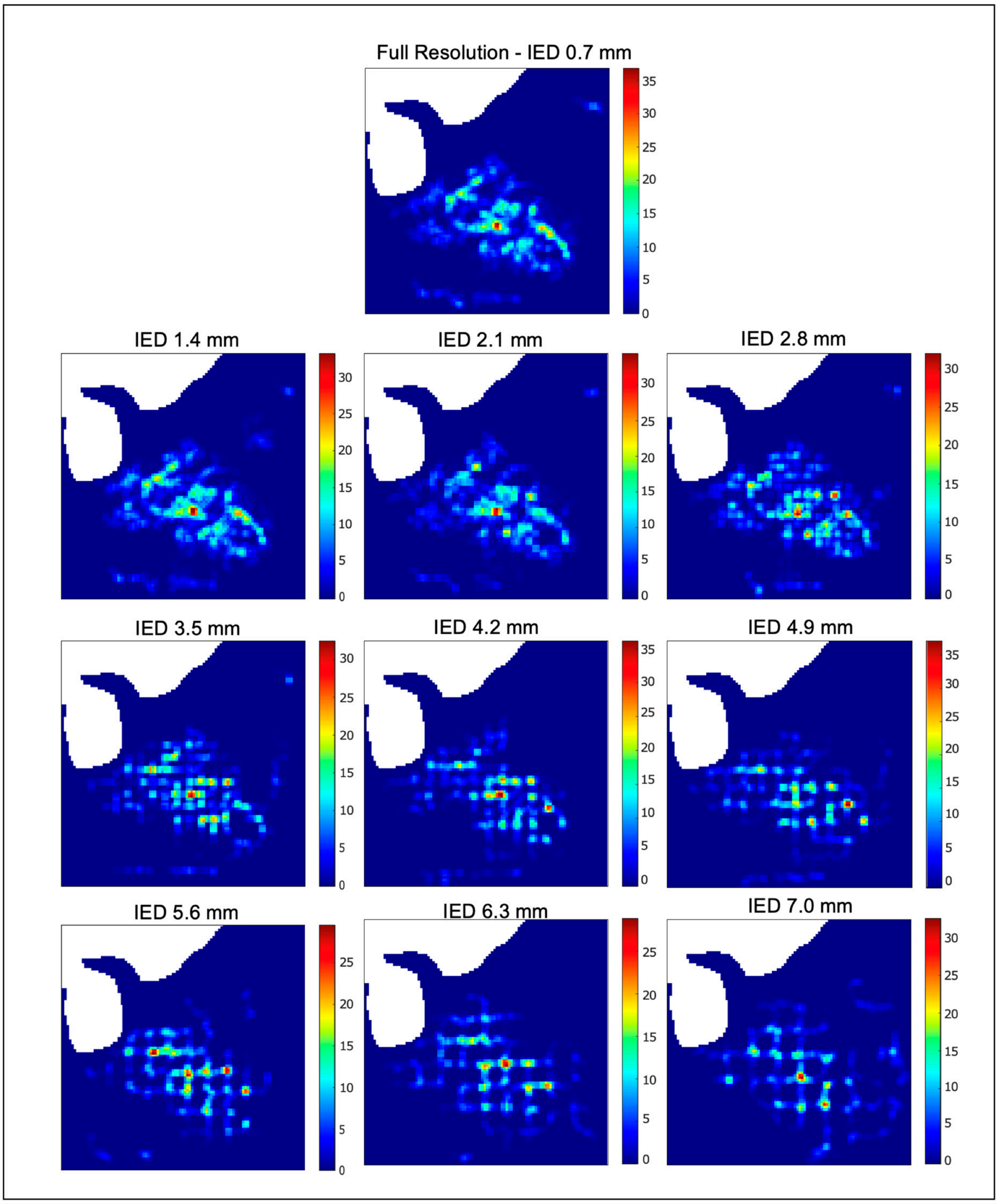

Supplementary Figure S18. Density maps

Rotor density maps of a single ventricular fibrillation recording at different spatial resolutions. IED = inter-electrode distance.

Supplementary Figure S19

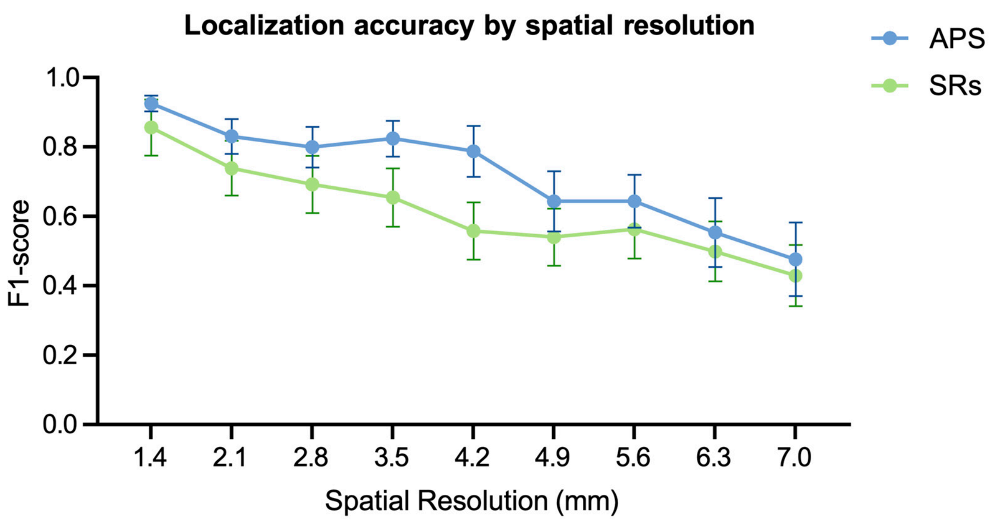

(a) All phase singularities (N=12).

IED (mm)	F1-score	CI
1.4	0.93 ± 0.02	0.88 - 0.99
2.1	0.84 ± 0.04	0.75 - 0.93
2.8	0.81 ± 0.05	0.71 - 0.91
3.5	0.79 ± 0.04	0.70 - 0.88
4.2	0.73 ± 0.07	0.58 - 0.88
4.9	0.63 ± 0.07	0.47 - 0.80
5.6	0.64 ± 0.08	0.47 - 0.80
6.3	0.56 ± 0.07	0.41 - 0.71
7.0	0.46 ± 0.08	0.28 - 0.63

(b) Stable rotors (N=11).

IED (mm)	F1-score	CI
1.4	0.91 ± 0.03	0.85 - 0.98
2.1	0.80 ± 0.04	0.70 - 0.89
2.8	0.74 ± 0.06	0.62 - 0.86
3.5	0.71 ± 0.06	0.58 - 0.85
4.2	0.61 ± 0.07	0.46 - 0.75
4.9	0.57 ± 0.07	0.41 - 0.72
5.6	0.62 ± 0.07	0.47 - 0.77
6.3	0.52 ± 0.08	0.36 - 0.69
7.0	0.44 ± 0.08	0.26 - 0.62

Supplementary Figure S19. Localization accuracy of PS density peaks

(Top) Comparison of localization accuracy, given by the F1-scores of the rotor density peaks, for all phase singularities (APS) and stable rotors (SRs) (n =11). (Bottom) F1-scores of the rotor density peaks and the respective confidence interval for each spatial resolution, for all data sets. F1-scores are presented as means±SEMs. IED = inter electrode distance, CI = confidence interval of the mean.

Supplementary Figure S20

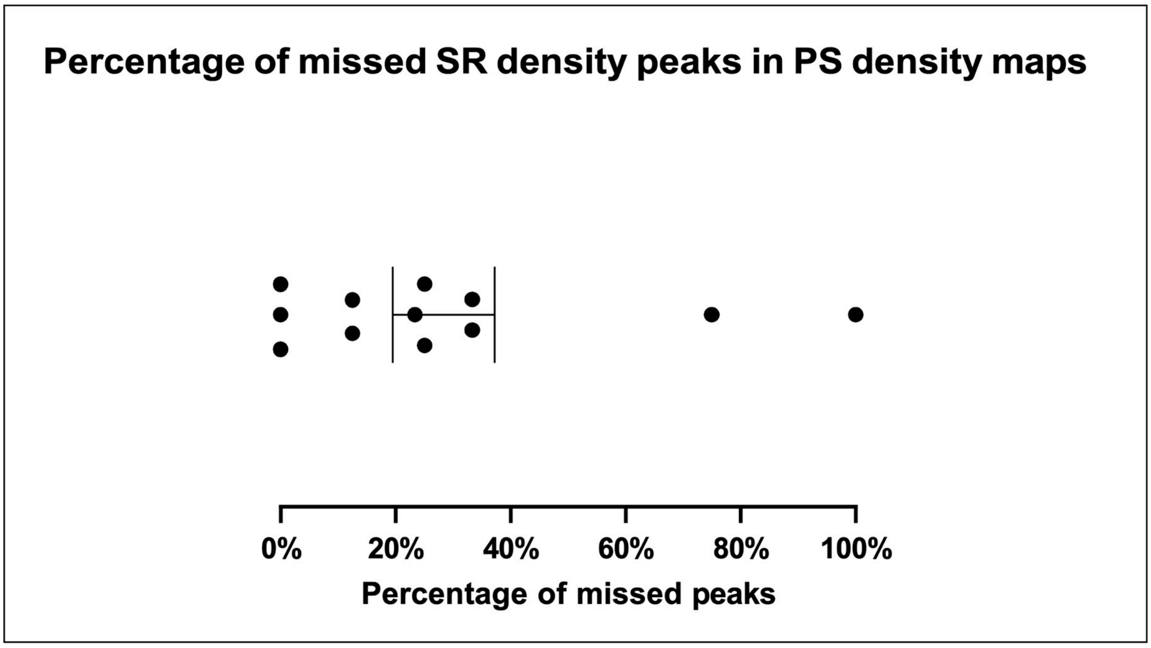

Supplementary Figure S20. Percentage of missed SR density peaks

Percentage of stable rotor (SR) density peaks missed in density maps with all phase singularities (APS). Each dot represents the mean for each heart data set. Vertical whiskers represent the standard error of the mean (n = 12).

Supplementary Figure S21

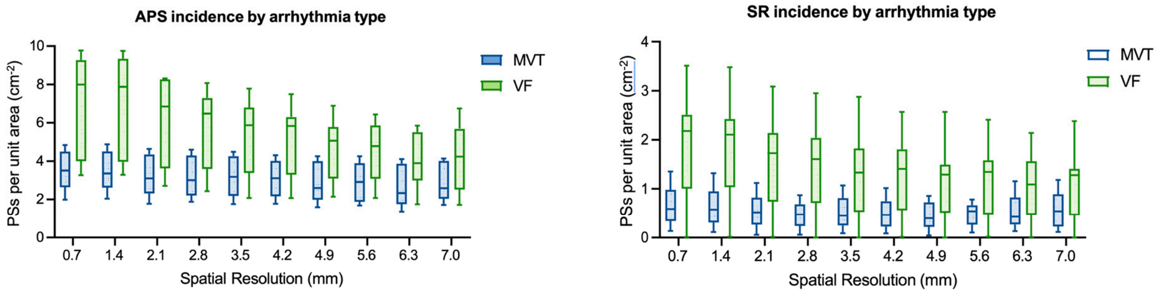

Source of Variation	*p* value	*p* value summary
Spatial resolution	0.0007	***
Arrhythmia type	0.0662	ns
Spatial resolution x Arrhythmia type	<0.0001	****

ns, not significant.
*** *p* value ≤ 0.001; **** *p* value ≤ 0.0001.

Source of Variation	*p* value	*p* value summary
Spatial resolution	0.0054	**
Arrhythmia type	0.0673	ns
Spatial resolution x Arrhythmia type	<0.0001	****

ns, not significant.
** *p* value ≤ 0.01; **** *p* value ≤ 0.0001.

Supplementary Figure S21. Rotor incidence by arrhythmia type

(Top) Comparisons of all phase singularities (APS) and stable rotor (SR) incidences for different arrhythmia types. (Bottom) Results of two-way repeated-measures ANOVA to assess the mean difference for APS and SR for various spatial resolutions and arrhythmia types (n =12). MVT = monomorphic ventricular tachycardia, VF = ventricular fibrillation.

Supplementary Figure S22

(a) MVT (N=5).

Comparison	Summary	*p* value
0.7 vs. 1.4	ns	0.9565
0.7 vs. 2.1	ns	0.2117
0.7 vs. 2.8	ns	0.3299
0.7 vs. 3.5	ns	0.2911
0.7 vs. 4.2	ns	0.1289
0.7 vs. 4.9	*	0.0394
0.7 vs. 5.6	ns	0.1228
0.7 vs. 6.3	*	0.0156
0.7 vs. 7.0	ns	0.0629

ns, not significant.

* p value ≤ 0.05.

(b) VF (N=7).

Comparison	Summary	*p* value
0.7 vs. 1.4	ns	0.4901
0.7 vs. 2.1	**	0.0038
0.7 vs. 2.8	*	0.0126
0.7 vs. 3.5	**	0.0069
0.7 vs. 4.2	*	0.0136
0.7 vs. 4.9	*	0.0118
0.7 vs. 5.6	*	0.0165
0.7 vs. 6.3	*	0.0159
0.7 vs. 7.0	*	0.0124

ns, not significant.

* p value ≤ 0.05; ** p value ≤ 0.01.

Supplementary Figure S22. Rotor incidence statistical summary by arrhythmia type for all phase singularities

Results of Dunnett's multiple-comparisons tests for mean differences between values for PS incidence at subresolutions and at ground truth for monomorphic ventricular tachycardia (MVT) recordings (a), and ventricular fibrillation (VF) recordings (b).

Supplementary Figure S23

(a) MVT (N=5).

Comparison	Summary	*p* value
0.7 vs. 1.4	ns	0.0714
0.7 vs. 2.1	ns	0.1450
0.7 vs. 2.8	ns	0.2858
0.7 vs. 3.5	ns	0.1555
0.7 vs. 4.2	ns	0.1541
0.7 vs. 4.9	ns	0.3007
0.7 vs. 5.6	ns	0.5912
0.7 vs. 6.3	ns	0.1035
0.7 vs. 7.0	ns	0.5386

ns, not significant.

(b) VF (N=6).

Comparison	Summary	*p* value
0.7 vs. 1.4	ns	0.5317
0.7 vs. 2.1	*	0.0204
0.7 vs. 2.8	*	0.0382
0.7 vs. 3.5	*	0.0237
0.7 vs. 4.2	*	0.0374
0.7 vs. 4.9	*	0.0259
0.7 vs. 5.6	*	0.0317
0.7 vs. 6.3	*	0.0350
0.7 vs. 7.0	*	0.0353

ns, not significant.
* p value ≤ 0.05.

Supplementary Figure S23. Rotor incidence statistical summary by arrhythmia type for stable rotors

Results of a Dunnett's multiple-comparisons tests for mean differences between values for stable rotor incidence at subresolutions and at ground truth for monomorphic ventricular tachycardia (MVT) recordings (a), and ventricular fibrillation (VF) recordings (b).

Supplementary Figure S24

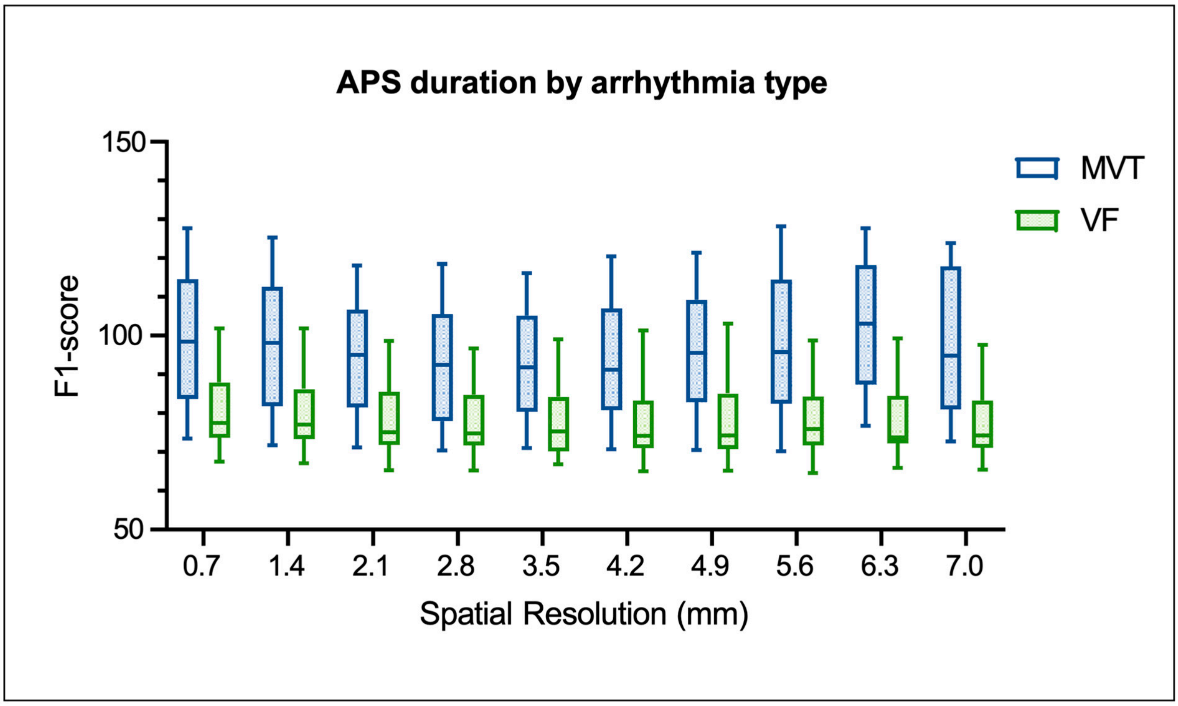

Supplementary Figure S24. Rotor duration by arrhythmia type for APS

Comparison of the durations of all phase singularities (APS) for different arrhythmia types (n=6 VF, n=5 MVT). MVT = monomorphic ventricular tachycardia, VF = ventricular fibrillation.

Supplementary Figure S25

Source of Variation	P value	P value summary
Spatial resolution	<0.0001	****
Arrhythmia type	0.8769	ns
Spatial resolution x Arrhythmia type	0.0011	**

ns, not significant.
** p value ≤ 0.01; **** p value ≤ 0.0001.

Supplementary Figure S25. Rotor detection accuracy statistical summary

Results of two-way repeated-measures ANOVA test to assess mean differences between F1-scores for various spatial resolutions and arrhythmia type (n=11)

Supplementary Figure S26

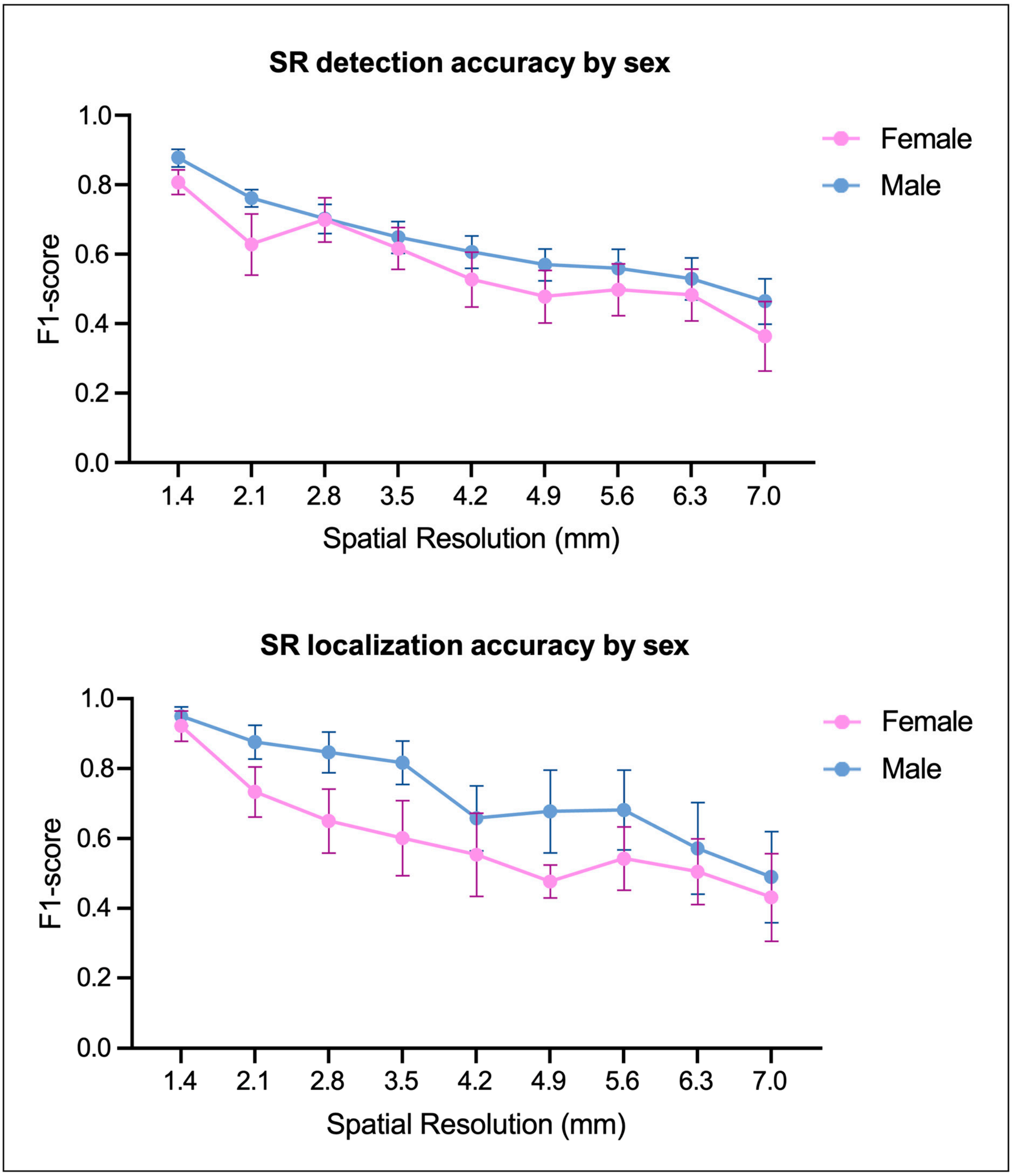

Supplementary Figure S26. Stable rotor detection and localization accuracy by sex

Comparison of stable rotor (SR) detection accuracy (top) and localization accuracy (bottom) for each sex given by F1-score of rotor density peak detection (n=6 males, 5 females). F1-scores presented as means±SEMs

Supplementary Figure S27

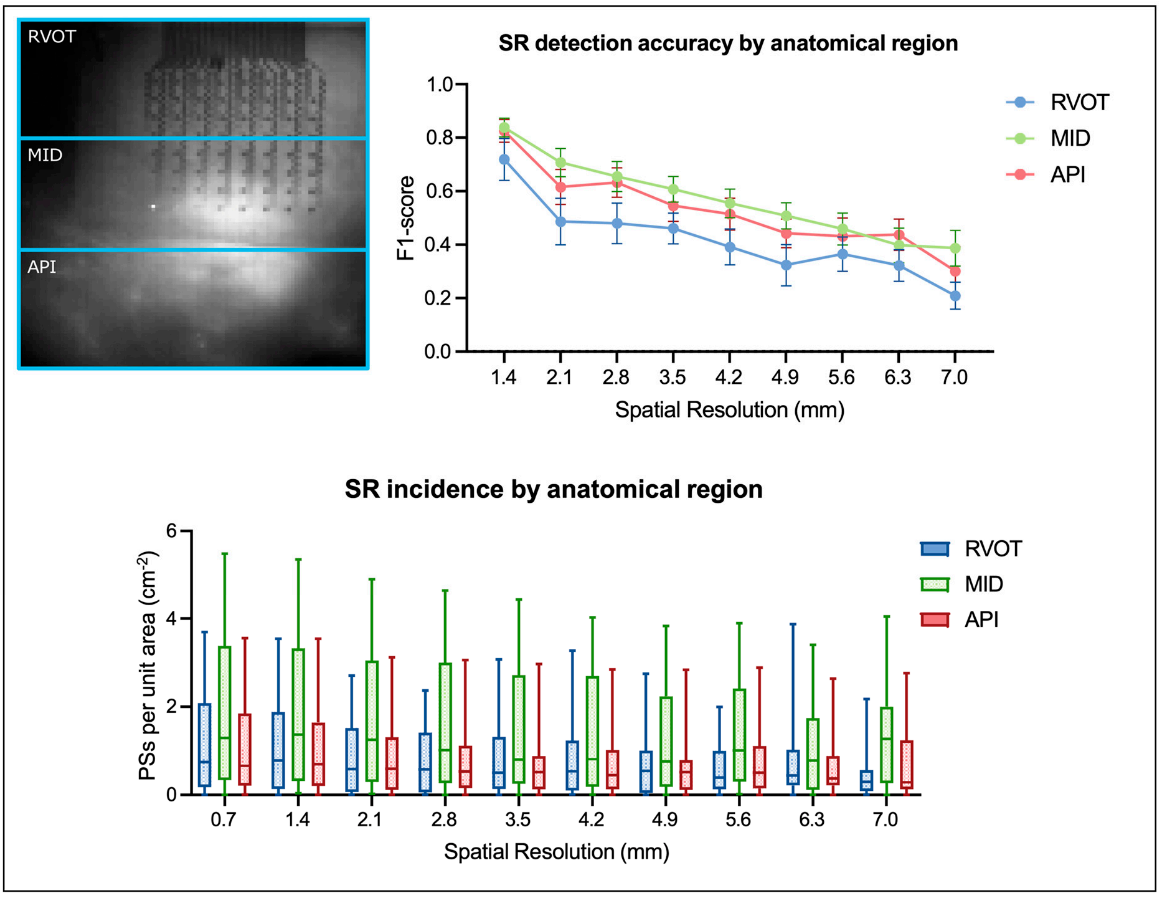

Supplementary Figure S27. Stable rotor incidence and detection by anatomical region

Comparison of stable rotor (SR) detection (top) and incidence (bottom) for different anatomical regions (n=11). RVOT = right ventricular outflow tract, MID = right ventricular mid region, API = right ventricular apical region.

Supplementary Figure S28

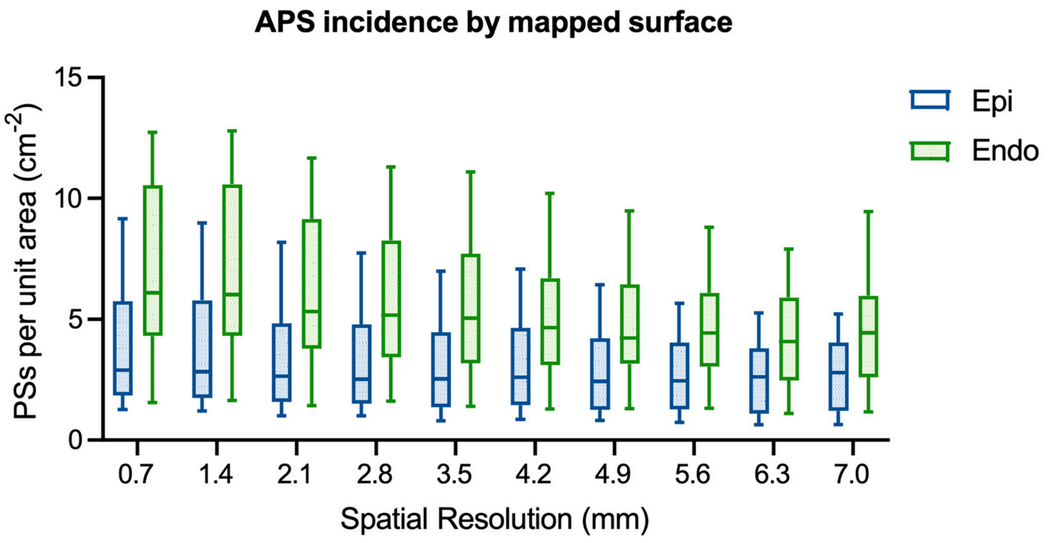

Source of Variation	*p* value	*p* value summary
Spatial resolution	<0.0001	****
Heart surface	0.0160	*
Spatial resolution x Heart surface	<0.0001	****

* p value ≤ 0.05; **** p value ≤ 0.0001.

Supplementary Figure S28. APS incidence by mapped surface

(Top) Comparison of the incidence of all phase singularities (APS) in the epicardium (Epi) and the endocardium (Endo) of the right ventricle (n = 12). Results of the two-way repeated-measures ANOVA test to assess the mean differences between APS incidence for various spatial resolutions and cardiac surface.

Supplementary Figure S29

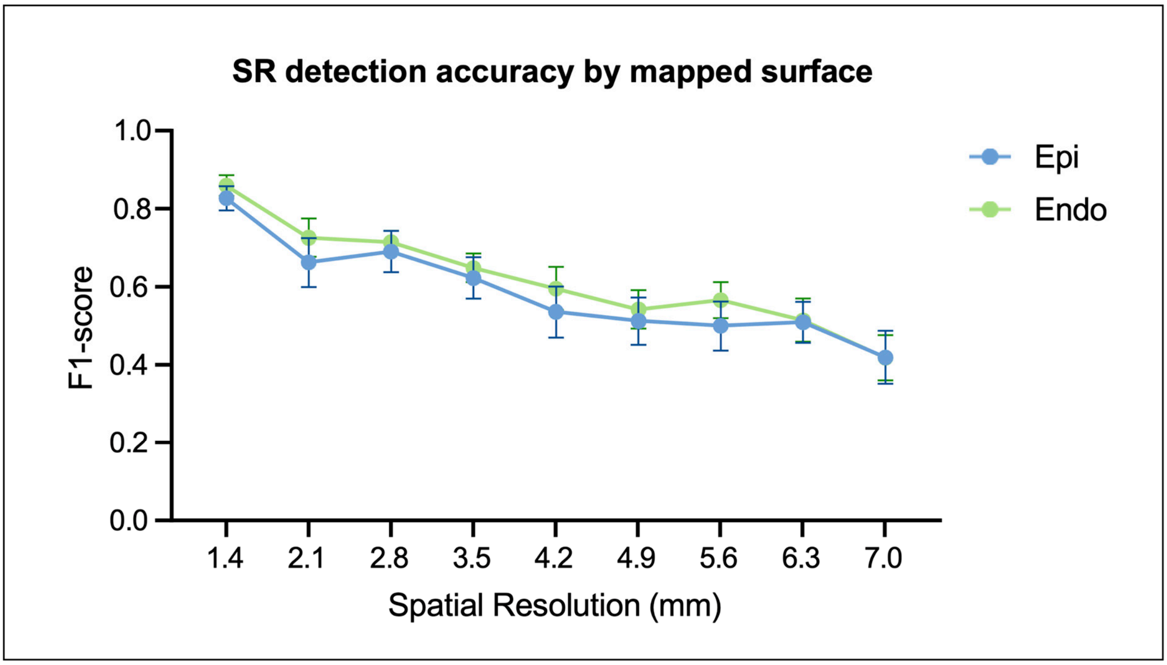

Supplementary Figure S29. Stable rotor detection accuracy by mapped surface

Comparison of stable rotor (SR) incidence in the epicardium (Epi) and the endocardium (Endo) of the right ventricle (n = 11), given by the F1-score of rotor detection. F1-scores presented as means ± SEMs

Supplementary Figure S30

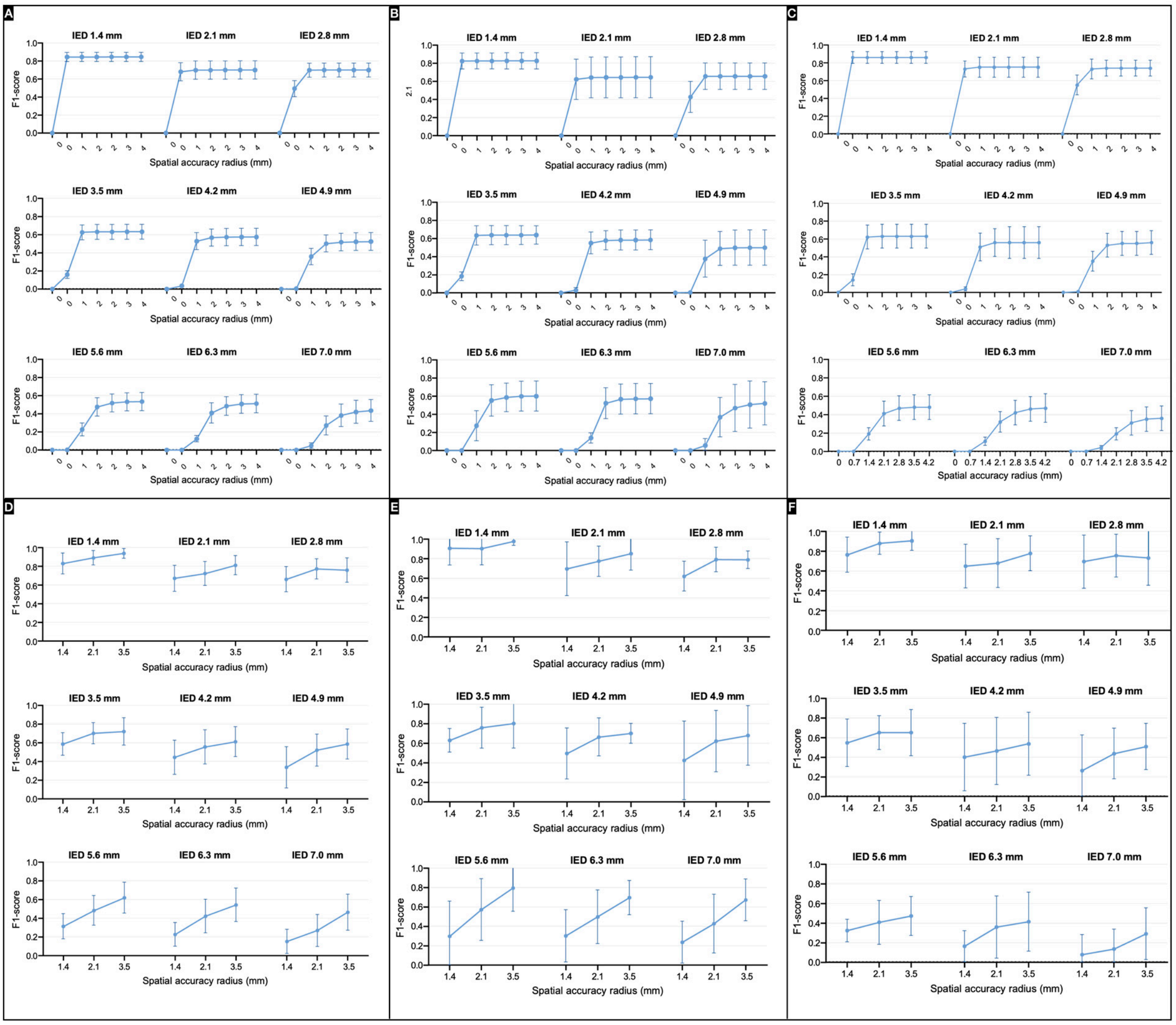

Supplementary Figure S30. Rotor detection accuracy statistical summary plots

Detection accuracy (F1-score) profile with 95% confidence intervals of the mean at each sub-resolution for all samples (n = 11) (A), for MVT (n = 5) (B), and for VF (n = 6) (C). Localization accuracy (F1-score) profile with 95% confidence intervals of the mean at each sub-resolution for all samples (D), for MVT (E), and for VF (F). IED = inter-electrode distance, MVT = monomorphic ventricular tachycardia, VF = ventricular fibrillation
